# Measuring progress in maternal and newborn health care in Mexico: validating indicators of health system contact and quality of care

**DOI:** 10.1186/s12884-016-1047-0

**Published:** 2016-08-30

**Authors:** Ann K. Blanc, Claudia Diaz, Katharine J. McCarthy, Karla Berdichevsky

**Affiliations:** 1 Population Council, New York, NY USA; 2 Population Council, Mexico City, Mexico

**Keywords:** Maternal and newborn health, Quality of care, Intervention coverage, Validation, Measurement, Indicators, Skilled birth attendance

## Abstract

**Background:**

The majority of births in Mexico take place in a health facility and are attended by a skilled birth attendant, yet maternal mortality has not declined to anticipated levels. Coverage estimates of skilled attendance and other maternal and newborn interventions often rely on women’s self-report through a population-based survey, the accuracy of which is not well established.

**Methods:**

We used a facility-based design to validate women’s report of skilled birth attendance, as well as other key elements of maternal, newborn intrapartum, and immediate postnatal care. Women’s reports of labor and delivery care were collected by exit interview prior to hospital discharge and were compared against direct observation by a trained third party in a Mexican public hospital (*n* = 597). For each indicator, validity was assessed at the individual level using the area under the receiver operating curve (AUC) and at the population level using the inflation factor (IF).

**Results:**

Five of 47 indicators met both validation criteria (AUC > 0.60 and 0.75 < IF < 1.25): urine sample screen, injection or IV medication received during labor, before the birth of the baby (i.e., uterotonic for either induction or augmentation of labor), episiotomy, excessive bleeding, and receipt of blood products. An additional 9 indicators met criteria for the AUC and 18 met criteria for the IF. A skilled attendant indicator had high sensitivity (90.1 %: 95 % CI: 87.1–92.5 %), low specificity (14.0 %: 95 % CI: 5.8–26.7 %) and was suitable for population-level estimation only.

**Conclusion:**

Women are able to give valid reports on some aspects of the content of care, although questions regarding the indication for interventions are less likely to be known. Questions that include technical terms or refer to specific time periods tended to have lower response levels. A key aspect of efforts to improve maternal and newborn health requires valid measurement of women’s access to maternal and newborn health interventions and the quality of such services. Additional work on improving measurement of population coverage indicators is warranted.

**Electronic supplementary material:**

The online version of this article (doi:10.1186/s12884-016-1047-0) contains supplementary material, which is available to authorized users.

## Background

The maternal mortality ratio in Mexico has declined by less than 7 % in the past two decades [1]. As part of its efforts to improve maternal health, the Mexican government has emphasized increasing access to skilled obstetric care as a central focus of the national health plan [2, 3]. To scale-up access, government-led initiatives have sought to promote contact with the health system and skilled birth attendance, establishing policies such as universal health coverage for pregnant women and no-cost emergency obstetric services [3].

To track such efforts, the Mexican government has emphasized monitoring women’s contact with the health system [4]. Indicators routinely collected as proxy measures for skilled obstetric care include the proportion of births delivered in health facilities and the proportion of births attended by a skilled birth attendant. In the absence of health monitoring systems that can provide accurate data on population coverage, these indicators often rely on women’s self-reports collected in household surveys such as the National Health and Nutrition Survey (Encuesta Nacional de Salud y Nutrición) in Mexico or international programs including the Demographic and Health Surveys (DHS) and Multiple Indicator Cluster Survey (MICS). However, the accuracy of women’s reports on these indicators is not well understood [5].

Insufficient progress in improving maternal health in Mexico and elsewhere has called into question reliance on indicators of health system contacts, rather than the content and quality of care. For example, researchers have noted that while physician-attended births were reported to be near universal in Mexico in 2012 (96 %), maternal deaths have not declined to the anticipated level [6–9].

A small, but emerging evidence base has sought to validate which aspects of maternal and newborn care women are able to report with accuracy. Validation research to date has largely taken place in settings where maternal mortality rates are low and often relies on hospital records, which often are subject to incomplete and inaccurate reporting as the reference standard. Although three rigorous studies were recently published in this area, to our knowledge, one of the most widely used indicators of health service contact – skilled attendance at birth– remains to be empirically validated [10–12]. In addition, apart from research by Tuncalp and colleagues which focused on validating women’s reports of cesarean section in the Dominican Republic [11], we know of little research to validate indicators of the quality and content of maternal and newborn health care in the Latin America and Caribbean region.

This study responds to the need for enhanced data related to the measurement of maternal and newborn health intervention coverage. We assessed the validity of a set of intrapartum and immediate postnatal indicators among women who delivered in a large Mexican public hospital. Our goal is to inform which aspects of intrapartum and immediate postnatal care women are able to report with accuracy and have the potential to be included in routine population-based surveys to monitor the coverage of lifesaving maternal and newborn health interventions.

As part of this work, a parallel study validated a set of quality of care indicators in Kenya [13]. Given variation in the status of maternal health and the organization of maternal health services, these results are reported separately.

## Methods

### Study design

Data for the validation analysis were drawn from women’s reports on the maternal and newborn health services they received during labor and delivery gathered by exit interviews prior to their discharge from the hospital. Women’s reports were compared against observations by a trained third party using a structured checklist. Direct observation was chosen as the reference standard as it was considered to yield the most accurate reflection of all facets of the care-giving process. In the event that clarification was needed (e.g., in a few instances the newborn and mother were taken into separate rooms and the observer remained with the mother) observations were supplemented by checking medical records or by consulting providers.

### Study setting and population

The study took place in a large public hospital in Mexico City where approximately 4,000 deliveries (54 % vaginal) take place annually. Hospital services include comprehensive obstetric care to women with normal pregnancies who are self-referred for admission, in combination with women with high-risk pregnancies who are referred from other public primary or secondary health care institutions. The population served by the study hospital tends to have a lower-than-average socioeconomic status and characteristically lacks health insurance. Patients may travel large distances to arrive at the study hospital with a large proportion from the neighboring State of Mexico (37 %) and 5 % from the rest of the country.

All pregnant women aged 15−49 who were admitted for delivery at the study hospital and were able to provide consent were eligible for participation. Women were excluded if they were unable to provide consent, if they presented with a complication, or if their stage of labor was considered too advanced by medical personnel. Study observation was discontinued if a cesarean section was indicated at any point.

### Ethical approval and consent

Written informed consent was obtained from all participants prior to observation. For women under the age of 18, written consent was also obtained from their spouse/ common law partner or parent (as responsible parties) in accordance with local ethical guidelines [14]. Provider consent was obtained to observe health care workers in the labor and delivery ward. A list of providers who gave their consent was provided to observers before they commenced observation. Very few providers refused participation.

The study protocol was approved by the Population Council’s Institutional Review Board and the Ethics and Research Committee of the participating hospital prior to participant enrollment.

### Data collection

Data collection took place between November 2013 and April 2014 and occurred over a 24-h period, using three daily observation shifts. Observations included all interactions between women and provider (s) upon admission to the labor ward until one hour following delivery. Client exit-interview questionnaires reflected the same elements of care and patient-provider interactions as the observation checklist (Additional files 1, 2, 3 and 4). Observers were general medical practitioners or nurses with sufficient clinical training to accurately record delivery room practices. Interviewers were social workers and psychologists with prior research experience. To minimize the potential for bias, study interviewers and observers were not the same individuals and all data collectors were external to the study facility. We recruited only female interviewers to facilitate rapport with participating women and interviews were conducted in Spanish. Data collectors received intensive training on the study protocol, including detailed explanation of each aspect of the client questionnaire and observation checklist to ensure full understanding, how to record responses, and the procedures for ethical research.

### Indicator selection

Indicators selected for validation were identified through a landscaping scan of published and grey literature conducted between April and July 2012, a detailed description of which is published elsewhere [13]. Inclusion criteria for indicator selection were: reflection of the content or quality of maternal and newborn health services relating to the early labor and immediate postnatal period (up to one hour following delivery) [15], current or proposed use in household survey programs (e.g., DHS or MICS) or the ability to reflect critical elements of care and be recalled by women. A total of 82 indicators were selected for validity testing (Additional file 5: Table S1).

Interview questionnaires were translated into Spanish and underwent minor modifications to improve participant understanding.

### Sample size

The target sample size for this study was 600 women. Since no information on the coverage of maternal and newborn interventions in the facility was available, we assumed 50 % prevalence for all indicators, with 60 % sensitivity ± 6 % precision, 70 % specificity ± 6 % precision, and with α = 0.05 assuming normal approximation to the binomial distribution. Using these assumptions, a target sample size of 500 was calculated. To allow for non-response, the sample size was increased to approximately 600 women.

#### Data analysis

Analyses were performed using Stata Version 12 (StataCorp, College Station, TX). We assessed two aspects of indicator validity, accuracy at the individual level and population level. To measure individual level reporting accuracy, we compared women and observer responses to a single or combination of questions computed for each indicator by constructing two-by-two tables. Missing and “Don’t Know” responses were excluded from the analysis. Where there was sufficient sample size (i.e., at least five counts per cell), we plotted the receiver-operating curve for each indicator. Receiver operating curve (ROC) analysis provides a global statistic of indicator accuracy by plotting the tradeoff between indicator sensitivity (i.e., the true positive rate), against its false positive rate (or 1 – specificity) [16]. To summarize the average accuracy of each indicator, we calculated the area under the receiver-operating curve (AUC). AUC scores range from 0 to 1, with an AUC of 0.5 representing a random guess and an AUC of 1 representing perfect diagnostic accuracy. For the purposes of this study we used an AUC of 0.6 or greater as an a priori benchmark of validity [12]. We present estimates of sensitivity, specificity and the AUC with accompanying 95 % confidence intervals (CIs) assuming a binomial distribution.

To estimate the prevalence that would be obtained for each indicator if assessed in a population-based survey (Pr), we applied the sensitivity (SE) and specificity (SP) calculated for each indicator to its true prevalence (P) (i.e., observer report) using the following equation: Pr = P x (SE + SP-1) + (1-SP) [17]. We then calculated the inflation factor (IF), or the ratio of the survey-based prevalence to the true prevalence, to estimate the degree to which each indicator would be over or under-estimated if assessed at the population level. A priori validation criteria for the IF was set at 0.75 < IF < 1.25 [12].

We defined overall acceptable indicator performance in terms of meeting both the individual (AUC) and population-level (IF) criteria (0.60 < AUC and 0.75 < IF < 1.25). However, we note that indicator validity criteria should be weighed according to the indicator’s intended use. For example, an indicator with low accuracy at the individual-level may produce an acceptable estimate of population-level coverage if the false positive reports and false negative reports yield a ratio of approximately one. Given this, we refer readers to the full validation results.

## Results

### Sample descriptive characteristics

**Fig. 1:**
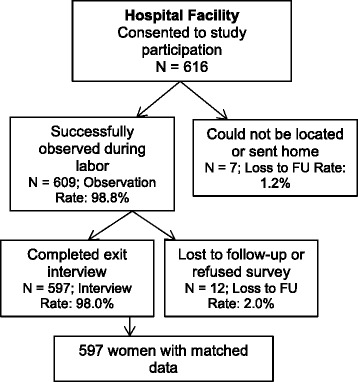
Participant response rates. Enrollment and response rates reflect women who delivered by vaginal birth only

In total, 779 women admitted for labor gave consent to participate. Due to hospital policies that allowed complete observation of women who underwent vaginal deliveries only, data collection for women who delivered by cesarean section was discontinued at the time of indication. Six hundred and sixteen women who consented to participate delivered by vaginal birth (Fig. 1). Of these women, 597 (97 %) were observed throughout labor and delivery and completed an exit interview before hospital discharge. The age, marital status, parity and education for women are presented in Table 1.

**Table 1:** Percent distribution of women by background characteristics

Variable	Percentage (%) *N* = 597
Age	
15-19	27.5
20-24	36.2
25-29	19.1
30-34	9.7
35-39	5.9
40+	1.7
Prior parity (Total number live births)	
0	47.7
1	29.3
2	14.4
3	5.4
4 or more	3.5
Educational attainment	
None	0.2
Primary	8.4
Secondary	42.3
Higher	49.2
Marital status	
Single, never married	25.6
Married	17.9
Living together	55.4
Separated	1.0
Widowed	0.0

### Validation results

The full list of indicators selected for validity testing as well as the prevalence of each indicator as reported by women and observers is presented in Additional file 5: Table S1. For all survey questions, women could respond “I Don’t Know”. Across indicators, “Don’t Know” responses were generally minimal. Greater than 5 % of women responded “Don’t Know” to 9 indicators (Table 2).

**Table 2:** Percentage of women who responded “Don’t Know” to survey questions

Client question	*N* ^a^	% “Don’t Know”
Immediately after the delivery of your baby, did anyone give you a medication or injection called oxytocin to help your uterus contract/become firm?	592	38.3
Did anyone give you medication or an injection called ‘oxytocin’ before you delivered the placenta?	414	52.9
While you were at the health facility for the birth of your baby, did you receive a HIV test?	596	23.7
Did you or anyone else give anything to the baby to eat or drink within the first hour after delivery?	593	21.4
Did the health provider(s) wash their hands with soap and water or use antiseptic before examining you?	592	19.4
Just after the delivery of your baby, in the first few minutes after the delivery of your baby, did anyone give you medication intravenously (through a through a tube in your arm)?	547	7.7
Was your baby dried off with a towel immediately, within a few minutes after his/her birth ?	593	6.6
About how long after birth was your baby bathed for the first time?	573	19.7
In your first physical examination/check after delivery, did a health provider check your belly to see whether your womb was becoming firm after the birth of your baby?	597	5.9

^a^Sample sizes vary by question due to the number of women who responded to each question. Note: In “Don’t Know” responses were excluded in the validation analysis. High ‘Don’t Know’ indicators were considered to be those questions to which greater than 5 % of women responded “I Don’t Know”

The highest percentage of women responded “Don’t Know” to questions regarding administration of oxytocin (i.e., uterotonic for the prevention of postpartum hemorrhage) at any time or before the delivery of the placenta (53 % and 38 %, respectively). An alternate proxy indicator regarding receipt of a uterotonic for prevention of postpartum hemorrhage – whether, immediately after delivery, “*anyone [gave] medication intravenously through a tube in your arm*” – also exceeded 5 % (8 %) “Don’t Know” responses. High percentages of “Don’t Know” responses were also recorded for several immediate postnatal interventions for the mother and newborn. In addition, nearly one-quarter of women did not know whether they received an HIV test. Anecdotal evidence from data collectors indicated that many women did not know what HIV was, or confused it with human papillomavirus (HPV).

There was adequate cell size to validate 47 indicators: five of which met both validation criteria (Table 3). These were: urine sample screen (i.e., check for preeclampsia), injection or IV medication received during labor and before the birth of the baby (i.e., proxy for uterotonic for either induction or augmentation of labor), episiotomy, hemorrhage, and receipt of blood products. An additional 27 indicators met either the criteria for individual-level classification (AUC) (9 indicators) or population-level coverage (IF) (18 indicators). We discuss the results below in relation to: (1) contact with the health system, (2) content and quality of maternal and immediate newborn health care, and (3) maternal health outcomes.

**Table 3:** Validation results for maternal and newborn intrapartum and immediate postnatal care indicators ^†^

Indicator	Total *N*	True Prev^a^ (%)	Sensitivity^b^ (95 % CI)	Specificity^c^ (95 % CI)	Self-report survey estimate (%), Based on sensitivity and specificity^d^	AUC (95 % CI) (>0.60)	IF^e^ (0.75 to 1.25)	Criteria Met^f^
*Indicators of Health Service Contacts*								
Main provider labor- doctor or medical resident	580	96.2	82.3 (78.8-85.3)	27.3 (10.7-50.2)	81.9	0.55 (0.51-0.59)	0.85	IF
Main provider delivery– doctor or medical resident	563	91.1	90.1 (87.1-92.5)	14.0 (5.8-26.7)	89.7	0.52 (0.48-0.56)	0.98	IF
*Indicators of Content of Care*								
Takes urine sample	572	25.2	50.0 (0.63-0.71)	84.4 (80.6-87.7)	24.3	0.67 (0.63-0.71)	0.97	Both
Injection or IV^g^ medication received at some time during labor, before birth of baby (general)	571	76.0	70.1 (65.5-74.3)	56.9 (48.2-65.4)	63.6	0.63 (0.59-0.68)	0.84	Both
Episiotomy performed	580	67.8	97.7 (95.7-98.9)	63.1 (55.8-70.0)	78.1	0.80 (0.77-0.84)	1.2	Both
Blood products given	582	2.41	35.7 (12.8-64.9)	98.4 (97.0-99.3)	2.4	0.67 (0.63-0.71)	1.0	Both
Receives injection or IV^g^ medication for induction or augmentation of labor	476	76.1	59.1 (53.9-64.2)	79.8 (71.3-86.8)	49.8	0.69 (0.65-0.74)	0.65	AUC
Augments labor with uterotonic	472	75.2	49.6 (44.3-54.9)	81.2 (72.9-87.8)	42.0	0.65 (0.61-0.70)	0.56	AUC
Membranes ruptured (labor induction or augmentation)	356	57.0	38.4 (31.7-45.5)	88.2 (82.0-92.9)	27.0	0.63 (0.58-0.68)	0.47	AUC
Breastfeeding initiated within first hour of birth^i^	449	34.7	81.4 (74.4-87.2)	44.1 (38.3-49.9)	64.8	0.63 (0.58-0.67)	1.9	AUC
First post-delivery exam, provider checks for involution	556	81.7	84.6 (80.9-87.8)	21.6 (14.0-30.8)	83.5	0.53 (0.49-0.57)	1.0	IF
Palpates uterus after delivery of placenta	580	82.4	79.5 (78.4-85.5)	19.6 (9.7-25.4)	79.7	0.50 (0.45-0.54)	0.97	IF
Uterotonic received 1-3 min after birth	580	64.0	61.5 (56.3-66.4)	40.7 (33.9-47.7)	60.7	0.51 (0.47-0.55)	0.95	IF
3 AMTSL^h^ elements: prophylactic uterotonic + controlled cord traction + uterine massage following delivery of placenta	549	81.2	75.7 (71.5-79.6)	24.0 (16.0-33.6)	75.7	0.50 (0.46-0.54)	0.93	IF
HIV status checked	569	69.8	54.9 (49.9-59.9)	47.7 (40.0-55.4)	54.1	0.51 (0.47-0.55)	0.78	IF
Induces labor with uterotonic	559	11.6	27.7 (17.3-40.2)	89.1 (86.0-91.7)	12.9	0.58 (0.54-0.62)	1.1	IF
Woman received pain relief medication	581	79.4	89.4 (86.2-92.0)	20.0 (13.3-28.3)	87.4	0.55 (0.51-0.59)	1.1	IF
First post-delivery exam, provider ask/checks for bleeding	590	80.5	87.4 (84.0-90.2)	12.2 (6.8-19.6)	87.5	0.50 (0.46-0.54)	1.1	IF
First post-delivery exam, provider examines perineum	586	75.1	90.2 (87.1-92.8)	10.3 (5.9-16.4)	90.1	0.50 (0.46-0.54)	1.2	IF
First post-delivery exam, provider takes temperature	586	66.9	94.4 (91.6-96.4)	8.8 (5.2-13.7)	93.4	0.52 (0.47-0.56)	1.4	
Encourages/assists woman to ambulate during labor	580	22.2	7.8 (3.8-13.8)	91.6 (88.6-94.0)	8.3	0.50 (0.46-0.54)	0.37	
Woman asked for pain relief medication at some time	575	44.7	32.7 (27.0-38.8)	78.9 (74.0-83.3)	26.3	0.56 (0.52-0.60)	0.59	
Something other than breastmilk given to baby within first hour of birth^i^	438	21.7	71.6 (61.4-80.4)	46.1 (40.7-51.5)	57.8	0.59 (0.54-0.64)	2.7	
Uterotonic received 1-3 min after delivery of placenta	576	20.0	95.7 (90.1-98.6)	6.3 (4.3-8.9)	94.1	0.51 (0.47-0.55)	4.7	
Baby given to mother immediately after birth^i^	580	10.3	63.3 (49.9-75.4)	40.6 (36.3-44.9)	59.8	0.52 (0.48-0.56)	5.8	
Maternal Complications								
Severe bleeding (hemorrhage)	593	8.1	50.0 (35.2-64.8)	95.2 (93.1-96.9)	8.4	0.73 (0.69-0.76)	1.0	Both
High blood pressure/convulsions (eclampsia)	594	2.4	57.1 (28.9-82.3)	79.5 (76.0-82.7)	21.4	0.68 (0.64-0.72)	9.1	AUC
Complication (yes to any)	595	13.6	60.5 (50.3-72.3)	65.4 (61.3-69.7)	38.2	0.64 (0.60-0.68)	2.8	AUC
None	595	86.4	65.6 (61.3-69.7)	61.7 (50.3-72.3)	61.8	0.64 (0.60-0.68)	0.72	AUC

^†^Validation analysis based on matched data, excluding ‘Don’t Know’ and missing responses. Sensitivity and specificity analysis was not performed for indicators that had fewer than 5 counts per cell of constructed two-by-two tables

^a^True prevalence refers to prevalence of intervention or event as measured by direct observation by a trained third party in the study hospital facility; ^b^True positive rate; ^c^True negative rate; ^d^Estimated prevalence that would be obtained through women’s self-report in a household survey (Pr), calculated using the following equation where *P* = true prevalence, SE = sensitivity, SP = specificity, Pr = P*(SE + SP-1) + (1-SP); ^e^IF = Pr/P, or ratio of survey-based prevalence to the true prevalence; ^f^AUC > 0.60 and 0.75 < IF < 1.25; ^g^IV refers to intravenous medication; ^h^Refers to active management of the third stage of labor; ^i^Questions asked of mothers whose infants were breathing at birth

*Note: For full description of study indicators, please see Additional file 5: Table S1

### Contact with the health system

Indicators of contact with the health system were: the type of provider involved in labor and delivery care and the type of facility where the delivery took place.

### Skilled birth attendance

To assess skilled birth attendance, women were asked, “*Who was the main provider assisting you during delivery?”* Cross-tabulation results of observer and women’s reports of provider type showed that most women reported attendance by a doctor (either a general provider or a doctor with specialization in obstetrics and gynecology (ob-gyn)), while most observers reported attendance by a medical resident (Table 4). In Mexico, there is little distinction between the type of care doctors and medical residents are legislated to provide. However, while doctors and medical residents are considered ‘skilled’, medical interns are not. To assess the accuracy of women’s reports of a ‘skilled’ attendant, we assessed a combined doctor and medical resident indicator. The true prevalence of skilled attendance during delivery (i.e., the main provider who ‘caught’ the baby was a doctor or medical resident) was 91 % and the reported prevalence was 90 %. This indicator had high sensitivity (90 %, 95 % CI: 87–93), low specificity (14 %, 95 % CI: 6–27 %) and met the IF criterion only (AUC: 0.52, 95 % CI: 0.48–0.56; IF 0.98).

**Table 4:** Cross-tabulation of women’s response and observer report of the main provider during delivery

Self- report (Number)	Observer report (Number)					
	Doctor /Ob-gyn	Medical resident	Medical intern	Nurse	Student nurse	Self-report total
Doctor /Ob-gyn	14	487	40	1	0	542
Medical resident	0	17	0	0	0	17
Medical intern	0	11	2	0	0	13
Nurse	0	11	0	1	0	12
Student nurse	0	0	0	0	0	0
Don’t Know	0	9	2	0	0	11
Observer Total	14	535	44	2	0	595

### Institutional delivery

Although this study was not designed to assess recall of the type of institution for delivery given that it took place in one facility only, for exploratory purposes women were asked to identify where they gave birth and whether it was a public or private sector facility. Replicating the methodology of the DHS and MICS surveys, women were also asked to specify the facility type (e.g., hospital, health clinic/center, health post, or other location). If women were unable to provide this information, they were asked to name the place.

Among women who responded to the question on whether the facility was classified as public or private sector and then specified its type, 85 % were correct. Among women who responded to the open-ended component of the question only: 83 % correctly named the facility, 9 % identified the facility as a hospital but did not identify the sector, and four percent identified both elements (e.g., reporting “Hospital público”, “Hospital federal”, or “Hospital de asistencia pública”); the open-ended probe elicits additional correct information not captured initially in the categorical response.

### Content of care

We validated 21 indicators related to interventions received throughout labor and the immediate postnatal period. Indicators of content of care with the highest individual-level accuracy were episiotomy (AUC: 0.80, 95 % CI: 0.77–0.84), followed by receiving an injection for the induction or augmentation of labor (AUC: 0.69, 95 % CI: 0.65–0.74), urine sample screen upon hospital admission, (AUC: 0.67, 95 % CI: 0.67–0.71) and receipt of blood products (AUC: 0.67, 95 % CI: 0.63 – 0.71). Indicators related to the third stage of labor, including receiving an injection or medication following delivery (i.e., uterotonic for the prevention of postpartum hemorrhage) and immediate postnatal care for the mother had moderate or low overall individual level validity.

Population-level accuracy for 13 content of care indicators were within 25 % of the true prevalence (0.75 < IF < 1.25). Overestimation was nearly double or greater the observed prevalence for four indicators: the infant was placed with the mother immediately after birth (5.8), receipt of an injection or medication following delivery of the placenta (i.e., uterotonic for the prevention of postpartum hemorrhage) (4.7), something other than breastmilk was given to the infant within the first hour of birth (2.7), and breastfeeding initiated in the first hour of birth (1.9).

### Uterotonic for prevention of postpartum hemorrhage

A critical component of maternal health care is receipt of a uterotonic for the prevention of hemorrhage, a leading cause of maternal death in Mexico and globally [18, 19]. Women were asked whether, “*Immediately after the delivery of your baby, did anyone given you”* (1) “*an injection in your thigh or buttocks*”, (2) “*medication intravenously (through a tube in your arm*”, or (3) *“tablets to swallow or hold in your mouth”, or* (4) “*tablets placed in your rectum”*. As the standard of care in the study facility was administration of the uterotonic via intravenous injection (100 % of cases observed), there was insufficient sample size to assess each of these indicators independently. Given this, we compared a combined indicator comprised of women who reported “Yes” to any method of uterotonic administration within the first few minutes of birth against the observer report of whether the woman received a uterotonic within three minutes of birth. The observed prevalence was 61 % and the reported prevalence was 64 %. The indicator did not meet the AUC (0.51, 95 % CI: 047–0.55) but did meet the IF criterion (0.95).

Women were asked the same set of questions about the first few minutes after the delivery of the placenta. A combined indicator constructed from women’s reports of whether she received a uterotonic via any method met neither criteria (AUC 0.51, 95 % CI: 0.47–0.55; IF 4.7). The specificity of this indicator (6 %, 95 % CI: 4–9 %) was low.

We attempted to validate an alternative indicator that used the name of the uterotonic drug and its purpose. Women were asked whether, “*Immediately after the birth of your baby, did anyone give you a medication or injection called oxytocin to help your womb contract/become firm?*” In the study facility, oxytocin was the standard uterotonic (administered in 97 % of cases). However, of women who reported “Yes” to any of the methods of uterotonic administration, 62 % responded either “Don’t Know” or “No” to the oxytocin question. While there was insufficient sample size to robustly analyze this indicator, descriptive results show that, among women who were observed to receive oxytocin and who gave a valid response to the question, 50 % accurately reported receiving the medication by its name.

### Maternal complications

Women were asked whether they had experienced a list of several obstetric complication symptoms (e.g., excessive bleeding, prolonged labor, high blood pressure, convulsions). There was sufficient sample size to assess the validity of four indicators based on these questions. One indicator- excessive bleeding (hemorrhage), met both acceptability criteria (IF 1.0, AUC 0.73, 95 % CI: 0.69–0.76). This indicator high specificity (95.2 %, 95 % CI: 93.1–96.9) but moderate sensitivity (50.0 %, 95 % CI: 35.2–64.8 %); nearly half of women who experienced excessive bleeding would not be captured in a survey question. The other three indicators met the individual criteria only and were overreported by women at the population level. For example, experiencing high blood pressure and convulsions (eclampsia) was overestimated by over 9 times at the population level (IF 9.1), although the low prevalence means that even slightly less than perfect reporting can lead to large overestimation.

## Discussion

Indicators that measure contact with the health system in addition to the quality of received care are critical for measuring progress in improving maternal and newborn health. This is the first study to empirically validate indicators related to the contact, quality and outcomes of maternal and immediate newborn postnatal care in Mexico. A strength of this study is the use of direct observation as the reference standard.

Few indicators assessed in the present study met both study validity criteria. This occurred in part because many interventions were routine practice and there was insufficient variation in the observed prevalence for robust analysis. However, an additional 9 indicators met the individual-level criteria and 18 met the population-level criteria. Indicators that did not meet both criteria are not necessarily invalid for all measurement purposes, but should be used in accordance with the rationale for their use. For example, indicators that do not meet criteria for the AUC but do meet the IF criterion may be suitable to measure intervention coverage at the population-level as false positive and negative reporting balance each other at the aggregate level.

Our findings also highlight the challenges of measuring low prevalence indicators accurately. Given that the calculation of the IF depends upon the indicator’s observed prevalence, even a small number of false positive responses can result in overestimation as measured by the IF. The implications of varying ‘true’ prevalence rates on the estimated prevalence that would be obtained from women in a household survey is illustrated in a previous article [13]. Additional validation work is needed to identify appropriate strategies for measuring low prevalence indicators, such as maternal complications and non-indicated practices.

The consistency of our results with prior findings is mixed. For example, the moderate sensitivity and high specificity for reported symptoms of hemorrhage (excessive bleeding) corresponds with levels found among women delivering in Indonesia, Benin and the Philippines [20–22]. However, these results differ from women’s reporting in Taiwan and Ghana [23]. We also found high sensitivity and low specificity for an indicator of whether or not the woman received a uterotonic within the first few minutes of birth; which differs from the low sensitivity and moderate specificity documented in a study conducted in Mozambique [12]. Taken together, these findings suggest that women’s understanding and recall of interventions received and obstetric complications experienced may vary by clinical and cultural context, and reinforces the importance of validating women’s reporting in different settings.

No contact with care indicators that we were able to assess (i.e., indicators of skilled birth attendance) met both validation criteria. However, a combined indicator of whether the primary birth attendant during delivery was a doctor/ob-gyn or medical resident did meet the criteria for valid estimation at the population level. Given that broad categorizations of provider types are likely to be more programmatically meaningful than the ability of women to delineate between finer categories of qualified health professionals (e.g., doctors versus medical residents), a composite indicator is likely to have utility for assessing population-level coverage. It is important to recognize, however, that skilled attendance is not synonymous with receiving appropriate care. Even a competent provider may not be able to provide high quality care if necessary supplies and other aspects of the enabling environment are inadequate [24, 25]. When possible, self-reported data should be triangulated with other data sources, such as stock-outs of essential medicines [26, 27].

Given that “skilled attendance” cannot be used reliably as a proxy for quality of care, indicators that measure the content of care administered by providers should also be assessed. Across content of care indicators assessed in this study, however, we observed a tendency for women to overreport some standard prevention practices, particularly related to the initial client assessment and immediate postnatal care for the mother and newborn. The high sensitivity and low specificity of many of these indicators suggests that in the few instances when an intervention was not administered, women tended to falsely report that it had occurred.

One potential explanation for this pattern is facility bias, or the assumption by women that they received quality care due to the fact that they delivered in a health facility [10, 13]. For example, more careful probing of some indicators of care, such as whether an injection or IV medication was received before birth (i.e., uterotonic for induction or augmentation of labor), or following delivery (i.e., uterotonic for prevention of postpartum hemorrhage) suggests that women did not know the specific indication for the care received, just that an intervention occurred. Validation results for the uterotonic indicators may reflect more general knowledge women had about having an IV line set, which was standard hospital practice for all women entering the labor and delivery ward, rather than knowledge of which medication was given or why. For example, neither an indicator of whether “an injection or IV medication was received to speed up labor” (i.e., uterotonic induction of labor) or whether “an injection or IV medication was received to strengthen labor” (i.e., uterotonic for augmentation of labor) met both validation criteria, while a general indicator of receiving an injection or IV medication at some time before the birth of the baby did. Similarly, most women correctly reported receipt of a uterotonic within the first few minutes after delivery of the baby, but also falsely reported the receipt of a uterotonic within the first few minutes after the delivery of the placenta, suggesting women may be able to report that some intervention occurred, but not be able to report accurately on aspects of its timing or purpose.

In light of the tendency for overreporting in a facility setting, high specificity for an indicator of receipt of a uterotonic may be favored at the cost of sensitivity. The near universal administration of oxytocin limited our ability to determine if an alternate uterotonic indicator that used the name of the drug and its purpose would achieve higher specificity as opposed to an indicator that asks women about the route of drug administration. Our descriptive results, however, did show that use of an indicator that uses the drug name captured substantially fewer true cases and fewer women were able to answer the question at all. This suggests that asking about receipt of a uterotonic using the drug name and reason for its use may not be a better alternative to asking women about whether they received an injection or some other method of drug administration.

An alternate explanation for positive reporting bias by women is that mothers misunderstood or conceptualized the meaning of questions differently than the observer definitions. For example, indicators with the highest “Don’t Know” responses generally included (1) technical terms, such as medication or disease names, related to the (2) timing of interventions received (including the sequence of newborn thermal care), or (3) the immediate postnatal period. Anecdotal evidence from study interviewers shows that additional explanation was needed to describe the difference between HIV vs. HPV, intravenous vs. intramuscular drug administration, and to define epidurals. Questions to which a substantial percentage of women responded “Don’t Know” (e.g. 20 % or more) are unlikely to yield valid information via self-reports and are not recommended for inclusion in population-based surveys.

Our findings highlight the importance of a two-part question with an open-ended component to improving reporting for complex indicators, such as the type of institution where the woman delivered. The fact that additional women could identify key facility attributes in an open-ended format suggests that providing the opportunity for women to name the specific facility, as is done in both the DHS and MICS surveys, is advantageous for capturing information on institutional deliveries.

While the study provides useful insight regarding the validity of contact and content of care indicators, there were some limitations. Due to budget limitations, the validation exercise took place in one type of facility only. Our study results are therefore reflective of women who delivered in a large public hospital. For example, although institutional delivery is near universal in Mexico (94 %), it is higher among women who reside in urban relative than rural areas (96 % and 88 %, respectively) and our results may be more reflective of this type of population [28]. The minimal variation in standard hospital procedures limited our ability to assess all indicators, which may have otherwise been proven valid. Additionally, in the study setting relatively few women experienced complications such as eclampsia, a leading cause of maternal death in Latin America and the Caribbean. It is possible that with a greater sample size, different combinations of questions regarding the symptoms of eclampsia could be validated to identify question wording with the highest accuracy.

Another limitation it that, due to hospital policy, it was not possible to include women who became indicated for a cesarean section delivery in the study. Future research should extend this work to a broader range of types of health facilities, including health centers and clinics, and may have the potential to validate the reporting accuracy of women who underwent a cesarean delivery. Validation research in other types of facilities may also reduce the potential for positive facility bias in reporting. Finally, exit interviews with women occurred shortly following delivery, prior to hospital discharge. The short recall time may cause women’s self-reports to be more accurate than in population level surveys such as the DHS and MICS when women are typically interviewed about a birth that took place one to three years prior. As part of an overall program of work on indicator validation, a separate follow-up study is underway to re-interview women approximately one year following delivery in Kenya.

## Conclusion

As attention in maternal health shifts to a new emphasis on ending preventable maternal mortality (EPMM) and addressing inequities, the tracking of progress will remain important [29]. A key aspect of these future efforts will require the valid measurement of not only *contact* with maternal and newborn health services but also *coverage* of key interventions that reflect the quality of services received. Until recently, however, little attention has been paid to improving the measurement of intervention coverage and quality at the population level. This information is needed to set programmatic priorities and to allocate resources effectively. Given the effect that this information can have on improvements in the lives of women and newborns, priority should be given to work that ensures that indicators of progress are selected, measured, and interpreted accurately.

## Additional files

Additional file 1: Client Exit Interview Questionnaire (English). (PDF 265 kb) Additional file 2: Client Exit Interview Questionnaire (Spanish). (PDF 1392 kb) Additional file 3: Observation Checklist (English). (PDF 280 kb) Additional file 4: Observation Checklist (Spanish). (PDF 1175 kb) Additional file 5: Table S1. List of Maternal and Newborn Care Indicators Assessed and Reported Prevalence. (DOC 173 kb)
