# Supplementary material for: Measuring progress in maternal and newborn health care in Mexico: validating indicators of health system contact and quality of care
Source: BMC Pregnancy Childbirth. 2016 Aug 30;16(1):255. doi: 10.1186/s12884-016-1047-0 (PMC5006493; doi:10.1186/s12884-016-1047-0)
Supplement: Additional file 1: — Client Exit Interview Questionnaire (English). (PDF 265 kb) [file 12884_2016_1047_MOESM1_ESM.pdf]

CLIENT EXIT QUESTIONNAIRE

Validating indicators of the quality of maternal health care

#	Question	Response codes	SKIP TO
**1. COVER SHEET INFORMATION**			
101.	INTERVIEWER NAME ID	|___|___|___|	
102.	FACILITY NAME ID	|___|___|___|	
103.	CLIENT ID	|___|___||___|___||___|___||___|___|	
104.	DATE OF INTERVIEW	|___|___||___|___||___|___| D D M M Y Y	
105.	OUTCOME OF INTERVIEW<br><br>**COMPLETE AT END OF INTERVIEW**	COMPLETED .... 1<br>PARTIALLY COMPLETED .... 2<br>REFUSED INTERVIEW .... 3<br>WOMAN WAS INCAPACITATED .... 4<br>PASSED AWAY 5	END<br>END<br>END<br>END<br>END
106.	TIME INTERVIEW BEGINS:<br>USE THE 24 HOUR CLOCK	HOURS |___|___| <br>MINUTES |___|___|	
107.	LANGUAGE USED TO CONDUCT INTERVIEW	ENGLISH .... 1<br>SPANISH / KISWAHILI.......... 2<br>LOCAL LANGUAGE (SPECIFY) .... 3<br>______________________	
108.	*Thank you for agreeing to speak to me today As you recall, you agreed to be interviewed about your birth. My questions should take 30 minutes at the most. If there is anything you do not understand, please do not hesitate to ask me questions.*<br><br>*A small gift of [NAME OF GIFT] will be provided to you today as thanks for your time, whether you agree or not to be interviewed.*<br>Any questions for me? Do I have your permission to continue?<br>| ___ | Yes | ___ | NO<br><br>Signature of interviewer:<br>(Indicates the agreement of the respondent to participate)<br><br>______________________________		

#	Question	Response codes	SKIP TO
	**2. BACKGROUND INFORMATION**		
201.	How old are you?<br><br>PROBE IF DK, OTHERWISE ESTIMATE AN AGE FOR THE WOMAN	AGE IN YEARS<br>|___|___|	
202.	What is the highest level of school you have attended?	NONE ....0<br>PRIMARY..1<br>SECONDARY ....2<br>HIGHER...3<br>DON'T KNOW..8	
203.	Are you now single, married, living together, divorced, separated or widowed?	SINGLE, NEVER MARRIED ....1<br>MARRIED ....2<br>LIVING TOGETHER.....................................3<br>SEPARATED ....4<br>DIVORCED...5<br>WIDOWED 6	
204.	To how many children have you given birth?	TOTAL N OF LIVE BIRTHS |____|____|	

#	Question	Response codes	SKIP TO
**3. LABOR AND DELIVERY**			
301.	**INTERVIEWER, RECORD WHETHER BABY IS ALIVE OR NOT**	YES .... 1<br>NO 0	
302.	What was the outcome for your delivery? Was your baby born alive or stillborn? **IF WOMAN DELIVERED TWINS, RECORD ANSWER FOR 2ND TWIN ONLY**	BORN ALIVE .... 1<br>STILLBORN 2	305
303.	Do you know, did your baby die before your labor pains started for your delivery?	YES .... 1<br>NO .... 0<br>DON'T KNOW 8	
304.	Were you shown your baby who was born?	YES .... 1<br>NO .... 0<br>DON'T KNOW 8	
305.	Did your labor start at home, en route to the health facility, once you were already at the hospital or did you never go into labor?	HOME .... 1<br>EN ROUTE TO FACILITY .... 2<br>ONCE AT FACILITY .... 3<br>NEVER WENT INTO LABOR .... 4<br>DON'T KNOW/CAN'T REMEMBER 8	
306.	Did you come to the facility because there was a problem while you were in labor or did you plan to deliver your baby in this facility?	YES, PROBLEM .... 1<br>NO, PLANNED TO COME .... 0<br>DON'T KNOW 8	
307.	Did you experience any of the following complications during or after your delivery?<br><br>CHECK ALL THAT APPLY	HIGH BLOOD PRESSURE/SEIZURES/BLURRED VISION/SEVERE HEADACHES .... 1<br>SWELLING IN HANDS/FEET .... 2<br>BABY WAS IN DISTRESS/TOO LARGE .... 3<br>LONG LABOR (MORE THAN 12 HOURS) ... 4<br>EXCESSIVE BLEEDING .... 5<br>INFECTION (FEVER) .... 6<br>OTHER COMPLICATIONS (SPECIFY) .... 7<br>________________<br>EXPERIENCED NO COMPLICATIONS .... 8<br>DK 9	

#	Question	Response codes	SKIP TO
308.	Can you tell me the type of facility where you gave birth to your baby (the category of this facility)?<br><br>*Probe to identify the type of source.*<br><br>*If unable to determine whether public or private sector, write the name of the place, according to the woman*<br><br>____________________<br>(Name of place)	PUBLIC SECTOR<br>GOVT. HOSPITAL....................................1<br>GOVT. CLINIC / HEALTH CENTRE ....2<br>GOVT. HEALTH POST ....3<br>OTHER PUBLIC SECTOR ....4<br>(*SPECIFY*)____________________<br><br>PRIVATE MEDICAL SECTOR<br>PRIVATE HOSPITAL.................................5<br>PRIVATE CLINIC ....6<br>PRIVATE MATERNITY HOME ....7<br>OTHER PRIVATE SECTOR ....8<br>(*SPECIFY*)____________________<br><br>OTHER PRIVATE SECTOR ....9<br>(*SPECIFY*)____________________	
309.	Did someone take your blood pressure, at or near your admission to the facility?	YES .... 1<br>NO .... 0<br>DON'T KNOW/CAN'T REMEMBER............. 8	
310.	Did someone ask you to give them a urine sample, at or near your admission to the facility?	YES .... 1<br>NO .... 0<br>DON'T KNOW/CAN'T REMEMBER............. 8	
311.	Did somebody listen to the heartbeat of the baby, at or near your admission to the facility?	YES .... 1<br>NO .... 0<br>DON'T KNOW/CAN'T REMEMBER............. 8	
312.	Did you receive an ultrasound test, at or near your admission to the facility?	YES .... 1<br>NO .... 0<br>DON'T KNOW/CAN'T REMEMBER............. 8	
313.	*Now I have some questions about HIV testing. However, please know in advance that we will not ask you your HIV status.*<br><br>During your time at this health facility for the birth of your baby, did someone ask you what your HIV status was?	YES .... 1<br>NO .... 0<br>DON'T KNOW/CAN'T REMEMBER............. 8	

#	Question	Response codes	SKIP TO
314.	While you were at this health facility for the birth of your baby, did someone offer you an HIV test?	YES .... 1<br>NO .... 0<br>DON'T KNOW/CAN'T REMEMBER 8	
315.	While you were at this health facility for the birth of your baby, did anyone test you for HIV?	YES .... 1<br>NO .... 0<br>DON'T KNOW/CAN'T REMEMBER 8	
316.	Who assisted with the delivery of your baby?<br><br>PROBE – *ANYONE ELSE?*<br><br>LIST ALL PROVIDERS PRESENT	YES / NO<br>DOCTOR (OBGYN) 1 2<br>MEDICAL RESIDENT 1 2<br>MEDICAL INTERN 1 2<br>NURSE 1 2<br>MIDWIFE 1 2<br>CLINCAL OFFICER 1 2<br>SUPPORT STAFF/AIDE 1 2<br>OTHER 1 2<br>(SPECIFY)<br>____________________	
317.	Who was the main provider(s) assisting you while you were in *labor*?<br><br>PROBE – *ANYONE ELSE?*<br><br>LIST ALL PROVIDERS PRESENT	YES / NO<br>DOCTOR (OBGYN) 1 2<br>MEDICAL RESIDENT 1 2<br>MEDICAL INTERN 1 2<br>NURSE 1 2<br>MIDWIFE 1 2<br>CLINCAL OFFICER 1 2<br>SUPPORT STAFF/AIDE 1 2<br>OTHER 1 2<br>(SPECIFY)<br>____________________	

#	Question	Response codes	SKIP TO
318.	Who was the main provider(s) who *actually delivered* your baby (caught the baby)?<br><br>PROBE – *ANYONE ELSE?*<br><br>LIST ALL PROVIDERS PRESENT	YES NO<br><br>DOCTOR (OBGYN) 1 2<br>MEDICAL RESIDENT 1 2<br>MEDICAL INTERN 1 2<br>NURSE 1 2<br>MIDWIFE 1 2<br>CLINCAL OFFICER 1 2<br>SUPPORT STAFF/AIDE 1 2<br>OTHER 1 2<br>(SPECIFY)<br><br>________________________________	
319.	How many providers in total assisted with the delivery of your baby?	|____|____|	
320.	For your birth of your baby, did your labor start spontaneously or did someone do something to bring on your labor?	SPONTANEOUS LABOR .... 1<br>SOMEONE DID SOMETHING...................... 2<br>DON'T KNOW/CAN'T REMEMBER............. 8	322<br><br>322
321.	For your birth of your baby, what was done to bring on your labor?	IV LINE INSERTED IN ARM........................ 1<br>MEMBRANES RUPTURED .... 2<br>OTHER (SPECIFY) .... 8<br><br>__________________________________	
322.	Were you allowed to have a companion with you during your labor and delivery?	YES.. 1<br>NO .... 0<br>DON'T KNOW/CAN'T REMEMBER............. 8	
323.	Did you have a companion with you during labor/delivery?	YES.. 1<br>NO .... 0<br>DON'T KNOW/CAN'T REMEMBER............. 8	 325<br>325

#	Question	Response codes	SKIP TO
324.	Who did you choose to accompany you during labor or delivery?	MOTHER........ 1<br>MOTHER-IN-LAW........ 2<br>SISTER........ 3<br>SISTER IN LAW........ 4<br>SPOUSE/PARNTER........ 5<br>OTHER FAMILY MEMBER........ 6<br>TRADITIONAL BIRTH ATTENDANT........ 7<br>OTHER (SPECIFY)........ 8<br>______________________	
325.	Were you allowed to get up and walk around while you were in labor?	YES........ 1<br>NO........ 0<br>DON'T KNOW/CAN'T REMEMBER........ 8	
326.	Did you choose to get up and walk around during labor?	YES........ 1<br>NO........ 0<br>DON'T KNOW/CAN'T REMEMBER........ 8	
327.	Were you allowed to drink liquids or eat any food while you were in labor?	YES........ 1<br>NO........ 0<br>DON'T KNOW/CAN'T REMEMBER........ 8	
328.	Did you choose to drink any liquids or eat food while you were in labor?	YES........ 1<br>NO........ 0<br>DON'T KNOW/CAN'T REMEMBER........ 8	
329.	For your delivery, was anything done to speed up or to strengthen your labor?	YES........ 1<br>NO........ 0<br>DON'T KNOW/CAN'T REMEMBER........ 8	 331<br>331
330.	What was done to speed up or to strengthen your labor?	RECEIVED INJECTION DURING LABOR........ 1<br>GIVEN IV MEDICATION DURING LABOR........ 2<br>MEMBRANES RUPTURED........ 3<br>OTHER (SPECIFY)........ 8<br>______________________	

#	Question	Response codes	SKIP TO
331.	For your delivery, did you receive any injections or IV medications during labor, that is, before the birth of your baby?	YES .... 1<br>NO .... 0<br>DON'T KNOW/CAN'T REMEMBER........ 8	
332.	Did the health provider(s) wash his/her hands with soap and water or use antiseptic before examining you?	YES .... 1<br>NO .... 0<br>DON'T KNOW/CAN'T REMEMBER........ 8	
333.	Did the health provider(s) wear rubber gloves during vaginal examinations?	YES, Always........ 1<br>YES, Sometimes .... 2<br>NO .... 0<br>DON'T KNOW/CAN'T REMEMBER........ 8	
334.	Did a health care provider ask you what position you wanted to choose during your labor OR for the delivery of your baby?	YES .... 1<br>NO .... 0<br>DON'T KNOW/CAN'T REMEMBER........ 8	
335.	What position were you actually in when you delivered your baby? That is, were you on your back, on your hands and knees, squatting or in another position?	ON YOUR BACK........ 1<br>HALF SITTING UP........ 2<br>LYING ON SIDE .... 3<br>ON HANDS AND KNEES .... 4<br>SQUATTING .... 5<br>OTHER (SPECIFY) .... 8<br>______________________	
336.	Was your baby delivered by cesarean, that is, did they cut your belly open to take the baby out?<br>**INTERVIEWER, EXPLAIN DIFFERENCE BETWEEN C-S AND EPISIOTOMY**	YES .... 1<br>NO .... 0<br>DON'T KNOW/CAN'T REMEMBER........ 8	 341<br>341
337.	Did you have your cesarean operation before you went into labor or after your labor had already started?	BEFORE LABOR STARTED........ 1<br>AFTER LABOR STARTED........ 2<br>DON'T KNOW, CAN'T REMEMBER........ 8	

#	Question	Response codes	SKIP TO
338.	What was the reason for your cesarean operation?	The doctor/nurse told me I had to............ 01<br>I was bleeding .... 02<br>The baby was stuck.................................... 03<br>I was in labor pain for a long time............. 04<br>The baby was not in the right position...... 05<br>I had a disease .... 06<br>My womb was broken/ruptured................ 07<br>There were problems with the baby.......... 08<br>There was no medical reason .... 09<br>Don't know 10	
339.	When was the decision for the cesarean taken?	BEFORE LABOR STARTED.......................... 1<br>AFTER LABOR STARTED............................. 2<br>DON'T KNOW, CAN'T REMEMBER............. 8	
340.	Who decided you would have a cesarean?	DOCTOR/PROVIDER.................................. 1<br>MYSELF.. 2<br>DON'T KNOW, CAN'T REMEMBER............. 8	
341.	For your delivery, shortly before you delivered your baby, did anyone cut the opening of your vagina (episiotomy) to make more room for the baby's head?	YES .... 1<br>NO .... 0<br>DON'T KNOW/CAN'T REMEMBER............. 8	
**CHECK RESPONSE TO 336. IF BABY DELIVERED BY CESAREAN SECTION, GO TO QUESTION 356.**			
342.	Did the health provider(s) wash his/her hands with soap and water or use antiseptic before delivering your baby?	YES .... 1<br>NO .... 0<br>DON'T KNOW/CAN'T REMEMBER............. 8	
343.	Did the health provider(s) wear rubber gloves during delivery of your baby?	YES, Always.. 1<br>YES, Sometimes .... 2<br>NO .... 0<br>DON'T KNOW/CAN'T REMEMBER............. 8	
344.	Did the health provider(s) wear rubber gloves while in handling the placenta?	YES, Always.. 1<br>YES, Sometimes .... 2<br>NO .... 0<br>DON'T KNOW/CAN'T REMEMBER............. 8	
Now I have some questions about you and your baby shortly after s/he was born.<br>(IF TWINS, REFER TO 2ND TWIN)			

#	Question	Response codes	SKIP TO
345.	*<u>Just after the delivery of your baby</u>* In the first few minutes after the delivery of your baby did anyone give you an injection in your thigh or buttock?	YES .... 1<br>NO .... 0<br>DON'T KNOW/CAN'T REMEMBER.......... 8	349
346.	*<u>Just after the delivery of your baby</u>* In the first few minutes after the delivery of your baby, did anyone give you medication intravenously (through a tube in your arm)?	YES .... 1<br>NO .... 0<br>DON'T KNOW/CAN'T REMEMBER.......... 8	349
347.	*<u>Just after the delivery of your baby</u>* In the first few minutes after the delivery of your baby did anyone give you tablets to swallow or hold in your mouth?	YES .... 1<br>NO .... 0<br>DON'T KNOW/CAN'T REMEMBER.......... 8	349
348.	*<u>Just after the delivery of your baby</u>* In the first few minutes after the delivery of your baby did anyone place tablets in your rectum?	YES .... 1<br>NO .... 0<br>DON'T KNOW/CAN'T REMEMBER.......... 8	
349.	*<u>Just after the delivery of your baby</u>* In the first few minutes after the delivery of your baby, did you receive a uterotonic/oxytocin *[insert local common name, e.g., pitocin]* to help your uterus contract/become firm?	YES .... 1<br>NO .... 0<br>DON'T KNOW/CAN'T REMEMBER.......... 8	
350.	**IF YES TO ANY OF ABOVE 5 QUESTIONS:**<br>Can you remember if you received this [injection/medication] BEFORE the delivery of the placenta?	YES .... 1<br>NO .... 0<br>PLACENTA DELIVERED IMMEDIATELY AFTER BABY .... 3<br>DON'T KNOW/CAN'T REMEMBER.......... 8	
351.	After the delivery of your baby, did the birth attendant help you deliver the placenta, that is, did he/she place his/her hand firmly on your lower abdomen with one hand and hold the umbilical cord in the other hand?	YES .... 1<br>NO .... 0<br>PLACENTA DELIVERED IMMEDIATELY AFTER BIRTH WITHOUT ASSISTANCE .... 3<br>DON'T KNOW/CAN'T REMEMBER.......... 8	

#	Question	Response codes	SKIP TO
352.	After the delivery of your baby, in the first few minutes *after the delivery of the placenta*, did anyone give you an injection in your thigh?	YES .... 1<br>NO .... 0<br>DON'T KNOW/CAN'T REMEMBER........ 8	355
353.	After the delivery of your baby, in the first few minutes *after the delivery placenta*, did anyone give you tablets to take or hold in your mouth?	YES .... 1<br>NO .... 0<br>DON'T KNOW/CAN'T REMEMBER........ 8	355
354.	After the delivery of your baby, did the birth attendant firmly massage your lower abdomen to help make your womb contract (become firm)?	YES .... 1<br>NO .... 0<br>DON'T KNOW/CAN'T REMEMBER........ 8	355
355.	After the delivery of the placenta, did the birth attendant firmly massage your lower abdomen to help make your womb contract (become firm)?	YES .... 1<br>NO .... 0<br>DON'T KNOW/CAN'T REMEMBER........ 8	
**CHECK QUESTIONS 301 and 302. IF OUTCOME IS STILLBIRTH, GO TO Q369**			
356.	Was your baby given to you immediately, within a few minutes, after his/her birth?	YES .... 1<br>NO .... 0<br>DON'T KNOW/CAN'T REMEMBER........ 8	
357.	Was your baby dried off with a towel immediately after his/her birth, within a few minutes of delivery?<br>IF TWINS, REFER TO 2ND TWIN	YES .... 1<br>NO .... 0<br>DON'T KNOW/CAN'T REMEMBER........ 8	
358.	Did someone place the baby on your chest, against your skin, immediately after delivery of the baby?<br>IF TWINS, REFER TO 2ND TWIN	YES .... 1<br>NO .... 0<br>DON'T KNOW/CAN'T REMEMBER........ 8	 361<br>361
359.	Was your baby wrapped in a cloth while lying against your chest or was your baby naked against your skin?	WRAPPED IN CLOTH .... 1<br>BABY NAKED AGAINST YOUR SKIN........ 2<br>DON'T KNOW/CAN'T REMEMBER........ 8	362<br><br>362
360.	(If naked against skin), was your baby covered with a towel or cloth while lying against your skin?	YES .... 1<br>NO .... 0<br>DON'T KNOW/CAN'T REMEMBER........ 8	ALL GO TO 362

#	Question	Response codes	SKIP TO
361.	Was your baby wrapped in a towel or cloth immediately after birth?	YES .... 1<br>NO .... 0<br>DON'T KNOW/CAN'T REMEMBER........ 8	
362.	Did anyone weigh your baby just after birth?<br>IF TWINS, REFER TO 2ND TWIN	YES .... 1<br>NO .... 0<br>DON'T KNOW/CAN'T REMEMBER........ 8	 365<br>365
363.	What was your baby's weight?<br>IF TWINS, REFER TO 2ND TWIN	WEIGHT IN GRAMS:<br>|___|___|___|___|	
364.	**INDICATE WHERE THE RESPONSE FOR WEIGHT CAME FROM**	WEIGHT TAKEN FROM HEALTH CARD....... 1<br>WEIGHT GIVEN BY MOTHER 2	
365.	Did you breastfeed your baby?<br>IF TWINS, REFER TO 2ND TWIN	YES .... 1<br>NO 0	 367
366.	Did you breastfeed your baby within the first hour after delivery?<br>IF TWINS, REFER TO 2ND TWIN	YES .... 1<br>NO .... 0<br>DON'T KNOW/CAN'T REMEMBER........ 8	
367.	Did you or anyone else give anything to the baby to eat or drink within the first hour after delivery?<br>IF TWINS, REFER TO 2ND TWIN	YES .... 1<br>NO .... 0<br>DON'T KNOW/CAN'T REMEMBER........ 8	
368.	About how long after birth was your baby bathed for the first time?<br>IF TWINS, REFER TO 2ND TWIN	WITHIN 1 HOUR........ 1<br>1-5 HOURS........ 2<br>6-24 HOURS .... 3<br>2-3 DAYS........ 4<br>AFTER 3 DAYS .... 5<br>DON'T KNOW/CAN'T REMEMBER........ 8	
369.	In your first physical examination/check after delivery, did a health provider look for or ask you about bleeding?	YES .... 1<br>NO .... 0<br>DON'T KNOW/CAN'T REMEMBER........ 8	
370.	Did you receive a blood transfusion at any point during your delivery or stay at the facility?	YES .... 1<br>NO .... 0<br>DON'T KNOW/CAN'T REMEMBER........ 8	

#	Question	Response codes	SKIP TO
371.	In your first physical examination/check after delivery, did a health provider do a perineal exam?	YES ..... 1<br>NO ..... 0<br>DON'T KNOW/CAN'T REMEMBER 8	
372.	In your first physical examination/check after delivery, did a health provider check your belly to see if your womb was becoming firm after the birth of your baby?	YES ..... 1<br>NO ..... 0<br>DON'T KNOW/CAN'T REMEMBER 8	
373.	In your first physical examination/check after delivery, did someone take your blood pressure?	YES ..... 1<br>NO ..... 0<br>DON'T KNOW/CAN'T REMEMBER 8	
374.	In your first physical examination/check after delivery, did someone take your temperature?	YES ..... 1<br>NO ..... 0<br>DON'T KNOW/CAN'T REMEMBER 8	
375.	Did you ask for pain relief at any point during your time at the health facility?	YES ..... 1<br>NO ..... 0<br>DON'T KNOW/CAN'T REMEMBER 8	
376.	Did anyone give you any pain relief at any point during your time at the health facility?	YES ..... 1<br>NO ..... 0<br>DON'T KNOW/CAN'T REMEMBER 8	
377.	Approximately how long after you were admitted to the facility did you deliver your baby?	1-3 HOURS ..... 1<br>3-6 HOURS ..... 2<br>6-12 HOURS ..... 3<br>12-24 HOURS ..... 4<br>MORE THAN 24 HOURS ..... 5<br>DON'T KNOW/CAN'T REMEMBER 8	
378.	Approximately how long from the time you delivered your baby did you stay in the facility?	1-6 HOURS ..... 1<br>6-12 HOURS ..... 2<br>12-24 HOURS ..... 3<br>1-2 DAYS ..... 4<br>2-3 DAYS ..... 5<br>AFTER 3 DAYS ..... 6<br>DON'T KNOW/CAN'T REMEMBER 8	

#	Question	Response codes	SKIP TO
379.	TIME INTERVIEW ENDS<br>1) **THANK THE RESPONDENT AND OFFER HER OUR GIFT**<br>2) **COMPLETE Q111**	HOURS |___|___| <br>MINUTES |___|___|	
