# Supplementary material for: Measuring progress in maternal and newborn health care in Mexico: validating indicators of health system contact and quality of care
Source: BMC Pregnancy Childbirth. 2016 Aug 30;16(1):255. doi: 10.1186/s12884-016-1047-0 (PMC5006493; doi:10.1186/s12884-016-1047-0)
Supplement: Additional file 2: — Client Exit Interview Questionnaire (Spanish). (PDF 1392 kb) [file 12884_2016_1047_MOESM2_ESM.pdf]

ID PACIENTE [___|___] [___|___] [___|___][___]

ANEXO II **CUESTIONARIO DE SALIDA A PACIENTES**

- Todas las preguntas se realizan esperando una respuesta espontánea a menos que se le indique que lea las posibilidades de respuesta.
- Para cada una de las preguntas a continuación, favor de marcar con una cruz en el recuadro que corresponda a la respuesta de la participante.
- En ocasiones se le pedirá que se salte algunas preguntas de este cuestionario. Cuando sea el caso, usted encontrará en la cuarta columna una nota que indica que pase a la siguiente pregunta con un flecha como la que se muestra a continuación:

302	¿Su trabajo (dolores) de parto comenzó de forma espontánea o alguien intervino (hizo algo) para que iniciara?	①.TRABAJO DE PARTO ESPONTÁNEO<br>②. ALGUIEN INTERVINO<br>⑧. NO SABE/ NO RECUERDA	①→322

No.	PREGUNTA	CÓDIGO DE RESPUESTA	PATRÓN DE SALTO
	**SECCIÓN 1: INFORMACIÓN DE LA CARÁTULA**		
101	CÓDIGO DEL ENTREVISTADOR	[__ __ __ __]	
102	CÓDIGO DEL SITIO DE OBSERVACIÓN	[__ __ __]	
103	CÓDIGO DE LA PACIENTE	[__ __ __| __ __ __|__ __ __]	
104	FECHA DE LA ENTREVISTA	[__ __] [__ __] [__ __] DD MM AA	
105	RESULTADO DE LA ENTREVISTA<br>✏ COMPLETAR UNA VEZ FINALIZADA LA OBSERVACIÓN	①.COMPLETADA<br>②. COMPLETADA PARCIALMENTE<br>③. RECHAZA ENTREVISTA<br>④. MUJER INCAPACITADA<br>⑤. ALTA VOLUNTARIA<br>⑥. MUJER FALLECIÓ	→FIN<br>→FIN<br>→FIN<br>→FIN<br>→FIN<br>→FIN
106	HORA EN LA QUE INICIA LA ENTREVISTA<br>✏ UTILICE EL RELOJ DE 24 HORAS	HORAS [___|___]<br>MINUTOS [___|___]	
107	LE AGRADEZCO HABER ACEPTADO PLATICAR HOY CONMIGO. COMO RECORDARÁ, USTED ACEPTÓ SER ENTREVISTADA SOBRE SU PARTO/CESÁREA. LAS PREGUNTAS QUE LE HARÉ TOMARÁN 30 MINUTOS COMO MÁXIMO. SI HAY ALGO QUE NO ENTIENDE, NO DUDE EN HACERME CUALQUIER PREGUNTA.<br><br>LE DAREMOS UN [VALE DE DESPENSA] DE REGALO COMO AGRADECIMIENTO POR SU TIEMPO, ACEPTE O NO SER ENTREVISTADA.<br>¿TIENE ALGUNA PREGUNTA? ¿TENGO SU PERMISO PARA CONTINUAR?<br><br>○ SÍ ○NO	....<br>Firma del entrevistador<br>(Indica que la entrevistada está de acuerdo en participar)	

TdP: Trabajo de parto

ANEXO II **CUESTIONARIO DE SALIDA A PACIENTES**

No.	PREGUNTA/ ACCIÓN	CÓDIGO DE RESPUESTA	SALTAR A
	**SECCIÓN 2: INFORMACION SOBRE ANTECEDENTES**		
201	¿Qué edad tiene?<br>✑ Sondear si no sabe.<br>✑ Estime una edad si es necesario.	[___|___] EDAD EN AÑOS	
202	¿Cuál es su nivel máximo de estudios?	⓪. NINGUNO<br>①. PRIMARIA<br>②. SECUNDARIA<br>③. PREPARATORIA/ ESCUELA TÉC.<br>④. UNIVERSIDAD<br>⑤. OTRO<br>ESPECIFICAR: ....<br>⑧. NO SABE	
203	¿Cuál es su estado civil?	①. SOLTERA, NUNCA SE HA CASADO<br>②. CASADA<br>③. EN UNIÓN LIBRE<br>④.SEPARADA<br>⑤. DIVORCIADA<br>⑥. VIUDA	
204	¿Cuántos hijos vivos ha tenido?	[___|___]<br>NÚMERO TOTAL DE HIJOS VIVOS	

CUESTIONARIO DE SALIDA A PACIENTES

➲ Proveedor de salud es sinónimo de personal de salud que atendió el trabajo de parto y parto en el Hospital General de México

No.	PREGUNTA/ ACCIÓN	CÓDIGO DE RESPUESTA	SALTAR A
	**SECCIÓN 3: TRABAJO DE PARTO Y EXPULSION**		
301	ENTREVISTADOR, REGISTRE SI EL BEBE ESTA VIVO O NO	①. SÍ ⓪. NO	
302	¿Cuál fue el resultado de su parto? ¿su bebé nació vivo o muerto?<br>✒ Si la mujer tuvo gemelos, registre la respuesta para el 2do gemelo solamente	①. NACIDO VIVO<br>②. ÓBITO (NACIÓ MUERTO)	①→304
303	¿Sabe si su bebé murió antes de comenzar con los dolores/contracciones/trabajo de parto?	①.SÍ ⓪.NO ⑧.NO SABE	
304	¿Le mostraron a su bebé cuando nació?	①.SÍ ②.NO ⑧.NO SABE	
305	¿Cuándo empezaron sus dolores de parto? ¿Empezaron en la casa, en camino al hospital, en el hospital?	①. CASA<br>②. DE CAMINO AL HOSPITAL<br>③. YA EN EL HOSPITAL<br>③. NUNCA INICIÓ TRABAJO DE PARTO<br>⑧. NO SABE/ NO SE ACUERDA	
306	¿Vino a este hospital porque se presentó algún problema durante su trabajo de parto o tenía planeado que su bebé naciera aquí?	①. SÍ, TUVE UN PROBLEMA<br>⓪. NO, PLANEABA VENIR<br>⑧. NO SABE	
307	¿Tuvo alguna de las siguientes complicaciones durante o después del parto?<br>✒ Posibilidad de varias respuestas. Lea y marque todas las que apliquen.	①. PRESION ALTA/ CONVULSIONES/VISION BORROSA/ DOLOR DE CABEZA INTENSO<br>②. PIES/MANOS HINCHADOS<br>③. SUFRIMIENTO FETAL /BEBE DEMASIADO GRANDE<br>④. PARTO DEMASIADO PROLONGADO (MAS DE 12 HORAS)<br>⑤. SANGRADO EXCESIVO<br>⑥. INFECCION (FIEBRE)<br>⑦. OTRAS COMPLICACIONES, ESPECIFICAR: ....<br>⑧. SIN COMPLICACIONES<br>⑨. NO SABE	

CUESTIONARIO DE SALIDA A PACIENTES

No.	PREGUNTA/ ACCIÓN	CÓDIGO DE RESPUESTA	SALTAR A
	**SECCIÓN 3: TRABAJO DE PARTO Y EXPULSION**		
308	¿Puede decirme en qué tipo de hospital nació su bebé?<br>➨ Sondear para identificar el tipo de institución.<br>➨ En caso de que la mujer no pueda determinar si es público o privado, anote el nombre del lugar de acuerdo a lo que reporte la mujer.<br>..<br>Nombre del lugar	**SECTOR PÚBLICO**<br>①. HOSPITAL DE GOBIERNO<br>②. CLÍNICA/ CENTRO DE SALUD DE GOBIERNO<br>③. PUESTO DE SALUD DEL GOBIERNO<br>④. OTRA INSTITUCIÓN DEL SECTOR PÚBLICO ESPECIFICAR: ....<br>**SECTOR PRIVADO**<br>⑤. HOSPITAL PRIVADO<br>⑥. CLÍNICA PRIVADA<br>⑦. CASA MATERNA PRIVADA<br>⑧. OTRA INSTITUCIÓN PRIVADA, ESPECIFICAR:	
309	¿Le tomaron la presión al momento, o poco después, de ser admitida al hospital?	①.SÍ<br>⓪.NO<br>⑧.NO SABE/ NO RECUERDA	
310	¿Le pidieron una muestra de orina al momento, o poco después, de ser admitida al hospital?	①.SÍ<br>⓪.NO<br>⑧.NO SABE/ NO RECUERDA	
311	¿Le revisaron los latidos del corazón del bebé al momento, o poco después, de ser admitida al hospital?	①.SÍ<br>⓪.NO<br>⑧.NO SABE/ NO RECUERDA	
312	¿Le hicieron un ultrasonido al momento, o poco después, de ser admitida al hospital?	①.SÍ<br>⓪.NO<br>⑧.NO SABE/ NO RECUERDA	
313	➨ Ahora le haré unas preguntas sobre VIH. Sin embargo, le aclaro de antemano que no le preguntaré si usted es VIH positiva<br>¿Durante su hospitalización para este parto, le preguntaron si tiene VIH?	①.SÍ<br>⓪.NO<br>⑧.NO SABE/ NO RECUERDA	
314	¿Durante su hospitalización para este parto, le ofrecieron hacerse una prueba de VIH?	①.SÍ<br>⓪.NO<br>⑧.NO SABE/ NO RECUERDA	
315	¿Durante su hospitalización para este parto, le realizaron una prueba de VIH?	①.SÍ<br>⓪.NO<br>⑧.NO SABE/ NO RECUERDA	

CUESTIONARIO DE SALIDA A PACIENTES

No.	PREGUNTA/ ACCIÓN	CÓDIGO DE RESPUESTA	SALTAR A
**SECCIÓN 3: TRABAJO DE PARTO Y EXPULSION**			
316	¿Cuál fue el PRINCIPAL PROVEEDOR DE SALUD que atendió EL TRABAJO DE PARTO?<br>✏ Esperar respuesta espontánea de la mujer<br>✏ Por favor especificar solo la persona principal que atendió el trabajo de parto.	①. MÉDICO<br>②. MÉDICO GINECÓLOGO<br>③. MÉDICO RESIDENTE<br>④. MÉDICO INTERNO<br>⑤. ESTUDIANTE DE MEDICINA<br>⑥. ENFERMERA<br>⑦. PASANTE DE ENFERMERÍA<br>⑧. ESTUDIANTE DE ENFERMERÍA<br>⑨. PARTERA<br>⑩. OTRO<br>ESPECIFICAR:	
317	¿Hubieron OTROS PROVEEDORES DE SALUD que atendieron EL TRABAJO DE PARTO?<br>✏ Esperar respuesta espontánea de la mujer<br>✏ Enliste todos los proveedores que atendieron el trabajo de parto aparte de la persona principal.	①. MÉDICO<br>②. MÉDICO GINECÓLOGO<br>③. MÉDICO RESIDENTE<br>④. MÉDICO INTERNO<br>⑤. ESTUDIANTE DE MEDICINA<br>⑥. ENFERMERA<br>⑦. PASANTE DE ENFERMERÍA<br>⑧. ESTUDIANTE DE ENFERMERÍA<br>⑨. PARTERA<br>⑩. OTRO<br>ESPECIFICAR:	
318	¿Cuál fue el PRINCIPAL PROVEEDOR DE SALUD que atendió EL PARTO?<br>✏ Esperar respuesta espontánea de la mujer<br>✏ Por favor especificar solo la persona principal que atendió el parto.	①. MÉDICO<br>②. MÉDICO GINECÓLOGO<br>③. MÉDICO RESIDENTE<br>④. MÉDICO INTERNO<br>⑤. ESTUDIANTE DE MEDICINA<br>⑥. ENFERMERA<br>⑦. PASANTE DE ENFERMERÍA<br>⑧. ESTUDIANTE DE ENFERMERÍA<br>⑨. PARTERA<br>⑩. OTRO<br>ESPECIFICAR:	
319	¿Hubieron otros PROVEEDORES DE SALUD que atendieron EL PARTO?<br>✏ Esperar respuesta espontánea de la mujer<br>✏ Enliste todos los proveedores que atendieron el parto aparte de la persona principal.	①. MÉDICO<br>②. MÉDICO GINECÓLOGO<br>③. MÉDICO RESIDENTE<br>④. MÉDICO INTERNO<br>⑤. ESTUDIANTE DE MEDICINA<br>⑥. ENFERMERA<br>⑦. PASANTE DE ENFERMERÍA<br>⑧. ESTUDIANTE DE ENFERMERÍA<br>⑨. PARTERA<br>⑩. OTRO<br>ESPECIFICAR:	

ANEXO II **CUESTIONARIO DE SALIDA A PACIENTES**

No.	PREGUNTA/ ACCIÓN	CÓDIGO DE RESPUESTA	SALTAR A
	**SECCIÓN 3: TRABAJO DE PARTO Y EXPULSION**		
320	¿Quién fue el PRINCIPAL PROVEEDOR DE SALUD que la atendió CUANDO NACIÓ SU BEBÉ (QUIÉN RECIBIÓ AL BEBÉ)?<br>➨ Esperar respuesta espontánea de la mujer.<br>➨ Por favor especificar solo la persona principal que la atendió cuando su bebé nació (quién recibió al bebé).	①. MÉDICO<br>②. MÉDICO GINECÓLOGO<br>③. MÉDICO RESIDENTE<br>④. MÉDICO INTERNO<br>⑤. ESTUDIANTE DE MEDICINA<br>⑥. ENFERMERA<br>⑦. PASANTE DE ENFERMERÍA<br>⑧. ESTUDIANTE DE ENFERMERÍA<br>⑨. PARTERA<br>⑩. OTRO<br>ESPECIFICAR:	
321	¿En total, cuántos proveedores de salud atendieron su parto?	[___|___]	
322	Su trabajo (dolores) de parto comenzó de forma espontánea o alguien intervino (hizo algo) para que iniciara?	①. TRABAJO DE PARTO ESPONTÁNEO<br>②. ALGUIEN INTERVINO<br>⑧. NO SABE/ NO RECUERDA	①→324
323	¿Qué le hicieron para que iniciara su trabajo de parto?	①. COLOCARON LÍNEA IV EN BRAZO<br>②. RUPTURA DE MEMBRANAS<br>⑧. OTRO, ESPECIFICAR:<br>..	
324	¿Le permitieron tener un acompañante durante el trabajo de parto y nacimiento de su bebé?	①. SÍ<br>⓪. NO<br>⑧. NO SABE/ NO SE ACUERDA	
325	¿Tuvo un acompañante durante el trabajo de parto y nacimiento de su bebé?	①. SÍ<br>⓪. NO<br>⑧. NO SABE/ NO SE ACUERDA	⓪→327
326	¿A quién eligió como acompañante durante el trabajo de parto y nacimiento de su bebé?<br>➨ Posibilidad de varias respuestas.	①. MADRE<br>②. SUEGRA<br>③. HERMANA<br>④. CUÑADA<br>⑤. ESPOSO/ PAREJA<br>⑥. OTRO MIEMBRO DE LA FAMILIA<br>⑦. PATERA TRADICIONAL<br>⑧. OTRO<br>ESPECIFICAR:	
327	¿Le permitieron pararse a caminar durante el trabajo de parto?	①. SÍ<br>⓪. NO<br>⑧. NO SABE/ NO SE ACUERDA	
328	¿Se paró a caminar durante el trabajo de parto?	①. SÍ<br>⓪. NO<br>⑧. NO SABE/ NO SE ACUERDA	

CUESTIONARIO DE SALIDA A PACIENTES

No.	PREGUNTA/ ACCIÓN	CÓDIGO DE RESPUESTA	SALTAR A
	**SECCIÓN 3: TRABAJO DE PARTO Y EXPULSION**		
329	¿Le permitieron tomar líquidos o comer algo durante el trabajo de parto?	①. SÍ<br>⓪. NO<br>⑧. NO SABE/ NO SE ACUERDA	
330	¿Tomó líquidos o comió algo durante el trabajo de parto?	①. SÍ<br>⓪. NO<br>⑧. NO SABE/ NO SE ACUERDA	
331	¿Le hicieron algo para acelerar o fortalecer sus dolores / el trabajo de parto para el nacimiento del bebé?	①. SÍ<br>⓪. NO<br>⑧. NO SABE/ NO SE ACUERDA	⓪→333
332	¿Qué le hicieron para acelerar o fortalecer sus dolores/ el trabajo de parto?	①. RECIBIÓ INYECCIÓN DURANTE TdP<br>②. MEDICAMENTO IV DURANTE EL TdP<br>③. RUPTURA DE MEMBRANAS<br>④. OTRO<br>ESPECIFICAR:.................................	
333	ANTES DE QUE NACIERA SU BEBE, ¿recibió alguna inyección o medicamento IV durante el trabajo de parto?	①. SÍ<br>⓪. NO<br>⑧. NO SABE/ NO SE ACUERDA	
334	¿Los proveedores de salud se lavaron las manos con agua y jabón o usaron antiséptico antes de revisarla?	①. SÍ<br>⓪. NO<br>⑧. NO SABE/ NO SE ACUERDA	
335	¿Los proveedores de salud utilizaron guantes de latex para realizarle tactos vaginales?	①. SÍ, SIEMPRE<br>②. SÍ, ALGUNAS VECES<br>⓪. NO<br>⑧. NO SABE/ NO RECUERDA	
336	¿Algún proveedor de salud le preguntó en qué posición quería estar usted durante el trabajo de parto o para el nacimiento de su bebé?	①. SÍ<br>⓪. NO<br>⑧. NO SABE/ NO SE ACUERDA	
337	¿En qué posición se encontraba para el parto? Es decir, ¿estaba acostada boca arriba, semi sentada, acostada de su lado izquierdo, sobre sus manos y rodillas, en cuclillas o en otra posición?	①. BOCA ARRIBA<br>②. SEMI-SENTADA<br>③. DECÚBITO LATERAL IZQUIERDO<br>④. SOBRE MANOS Y RODILLAS<br>⑤. CUCLILLAS<br>⑥. OTRA<br>ESPECIFICAR:	
338	¿Su bebé nació por cesárea? Es decir, ¿le abrieron por la vientre (panza) para sacar al bebé?<br>✒ Entrevistador(a), explique la diferencia entre cesarea y episiotomia	①. SÍ<br>⓪. NO<br>⑧. NO SABE/ NO SE ACUERDA	⓪→343

No.	PREGUNTA/ ACCIÓN	CÓDIGO DE RESPUESTA	SALTAR A
	**SECCIÓN 3: TRABAJO DE PARTO Y EXPULSION**		
339	¿Le hicieron la cesárea antes de que iniciara su trabajo de parto o después de que comenzó	①. ANTES DE INICIAR EL TdP<br>②. DESPUÉS DE INICIAR EL TdP<br>⑧. NO SABE/ NO SE ACUERDA	
340	¿Cuál fue la razón para que le hicieran la cesárea?	①. EL DOCTOR/ ENFERMERA ME DIJO QUE LA TENÍAN QUE HACER<br>②. ESTABA SANGRANDO<br>③. EL BEBÉ ESTABA ATORADO<br>④. LLEVABA DEMASIADO TIEMPO EN TdP<br>⑤. LA POSICIÓN DEL BEBÉ NO ERA CORRECTA<br>⑥. TENÍA UNA ENFERMEDAD<br>⑦. MI MATRIZ ESTABA ROTA<br>⑧. EL BEBÉ TENÍA PROBLEMAS<br>⑨. NO HABÍA RAZÓN MÉDICA<br>⑩. NO SÉ	
341	¿En qué momento se tomó la decisión de hacer la cesárea?	①. ANTES DE INICIAR EL TdP<br>②. DESPUÉS DE INICIAR EL TdP<br>⑧. NO SABE/ NO SE ACUERDA	
342	¿Quién decidió que se haría una cesárea?	①. DOCTOR/ PROVEEDOR<br>②. YO MISMA<br>⑧. NO SABE/ NO SE ACUERDA	
343	Para el parto, poco antes del nacimiento del bebé, ¿le cortaron la abertura de la vagina (episiotomía) para hacerle más espacio para la cabeza del bebé?	①. SÍ<br>⓪. NO<br>⑧. NO SABE/ NO SE ACUERDA	
	REVISE LA RESPUESTA 338. SI EL BEBÉ NACIO POR CESÁREA, SIGA A LA PREGUNTA 358.		
344	¿Los proveedores de salud se lavaron las manos con agua y jabón o usaron antiséptico antes de atender el parto?	①. SÍ<br>⓪. NO<br>⑧. NO SABE/ NO SE ACUERDA	
345	¿Los proveedores de salud utilizaron guantes de latex para atender el parto?<br>✏ En caso que la respuesta sea "Sí", ¿fue siempre o sólo algunas veces?	①. SÍ, SIEMPRE<br>②. SÍ, ALGUNAS VECES<br>⓪. NO<br>⑧. NO SABE/ NO RECUERDA	
346	¿Los proveedores de salud utilizaron guantes de latex para manipular la placenta?<br>✏ En caso que la respuesta sea "Sí", ¿fue siempre o sólo algunas veces?	①. SÍ, SIEMPRE<br>②. SÍ, ALGUNAS VECES<br>⓪. NO<br>⑧. NO SABE/ NO RECUERDA	

No.	PREGUNTA/ ACCIÓN	CÓDIGO DE RESPUESTA	SALTAR A
	**SECCIÓN 3: TRABAJO DE PARTO Y EXPULSION**		
	Ahora le haré algunas preguntas sobre usted y su bebé, inmediatamente después del nacimiento. (EN CASO DE GEMELOS, REFERIRSE AL 2do GEMELO)		
347	*Inmediatamente después del nacimiento de su bebé* ¿Alguien le puso una inyección en el muslo o en el glúteo (nalga) durante los primeros minutos después del parto?	①. SÍ<br>⓪. NO<br>⑧. NO SABE/ NO SE ACUERDA	①→351
348	*Inmediatamente después del nacimiento de su bebé* ¿Alguien le dio un medicamento intravenoso (por una manguerita en su brazo) durante los primeros minutos después del parto?	①. SÍ<br>⓪. NO<br>⑧. NO SABE/ NO SE ACUERDA	①→351
349	*Inmediatamente después del nacimiento de su bebé* ¿Alguien le dio unas pastillas para tragar o para mantener en su boca durante los primeros minutos después del parto?	①. SÍ<br>⓪. NO<br>⑧. NO SABE/ NO SE ACUERDA	①→351
350	*Inmediatamente después del nacimiento de su bebé* ¿Alguien le colocó unas pastillas por el recto durante los primeros minutos después del parto?	①. SÍ<br>⓪. NO<br>⑧. NO SABE/ NO SE ACUERDA	
351	*Inmediatamente después del nacimiento de su bebé* ¿Alguien le dio un medicamento llamado *oxitocina* para ayudar a que su matriz se contrajera / se endureciera?	①. SÍ<br>⓪. NO<br>⑧. NO SABE/ NO SE ACUERDA	
352	**SI CONTESTO SI A CUALQUIERA DE LAS 5 PREGUNTAS PREVIAS:** ¿Se acuerda si le dieron un medicamento / inyección llamada *oxitocina* ANTES de que expulsara la placenta?	①. SÍ<br>⓪. NO<br>③. PLACENTA SALIÓ INMEDIATAMENTE DESPUÉS DEL BEBÉ<br>⑧. NO SABE/ NO RECUERDA	
353	Después de que nació su bebé, ¿el proveedor le ayudó a que expulsara la placenta? Es decir, ¿el proveedor colocó una mano firmemente sobre su abdomen bajo mientras sostuvo el cordón umbilical con la otra mano?	①. SÍ<br>⓪. NO<br>③. PLACENTA SALIÓ INMEDIATAMENTE DESPUÉS DEL BEBÉ<br>③. NO SABE/ NO RECUERDA	
354	Después de que nació su bebé, ¿le pusieron una inyección en el muslo en los primeros minutos *después de que expulsó la placenta?*	①. SÍ<br>⓪. NO<br>⑧. NO SABE/ NO SE ACUERDA	①→356
355	Después de que nació su bebé, ¿le dieron unas pastillas para tragar o mantener en su boca, en los primeros minutos *después de que expulsó la placenta?*	①. SÍ<br>⓪. NO<br>⑧. NO SABE/ NO SE ACUERDA	

ANEXO II **CUESTIONARIO DE SALIDA A PACIENTES**

No.	PREGUNTA/ ACCIÓN	CÓDIGO DE RESPUESTA	SALTAR A
	**SECCIÓN 3: TRABAJO DE PARTO Y EXPULSION**		
356	Después del nacimiento de su bebé, ¿el proveedor de salud le hizo un masaje firme en su abdomen bajo para ayudar a que su matriz se endureciera / contrajera?	①. SÍ<br>⓪. NO<br>⑧. NO SABE/ NO SE ACUERDA	
357	Después de la expulsión de la placenta, ¿el proveedor de salud le hizo un masaje firme en su vientre bajo para ayudar a que su matriz se endureciera / contrajera?	①. SÍ<br>⓪. NO<br>⑧. NO SABE/ NO SE ACUERDA	
	**REVISAR LAS PREGUNTAS 301 y 302. SI LA RESPUESTA ES OBITO (BEBÉ NACIÓ MUERTO), SALTAR A LA PREGUNTA 371**		
358	¿Le entregaron a su bebé de forma inmediata después del parto / a los pocos minutos del nacimiento?<br>✎ En caso de gemelos, referirse al 2do gemelo	①. SÍ<br>⓪. NO<br>⑧. NO SABE/ NO SE ACUERDA	
359	¿Secaron a su bebé con una toalla (o tela, campo) inmediatamente después del parto / a los pocos minutos del nacimiento?<br>✎ En caso de gemelos, referirse al 2do gemelo	①. SÍ<br>⓪. NO<br>⑧. NO SABE/ NO SE ACUERDA	
360	¿Inmediatamente después del nacimiento, le colocaron al bebé sobre su pecho, en contacto con su piel?<br>✎ En caso de gemelos, referirse al 2do gemelo	①. SÍ<br>⓪. NO<br>⑧. NO SABE/ NO SE ACUERDA	⓪→363
361	Mientras tenía al bebé sobre su pecho, ¿estaba envuelto en una tela o lo tenía desnudo contra su piel?	①. ENVUELTO EN LA TELA<br>②. BEBÉ DESNUDO EN CONTACTO CON LA PIEL DE LA MADRE<br>⑧. NO SABE/ NO RECUERDA	①→363
362	*(Si estaba desnudo contra su piel)*, ¿Estaba el bebé cubierto con una toalla o tela mientras lo tenía sobre su pecho?	①. SÍ<br>⓪. NO<br>⑧. NO SABE/ NO SE ACUERDA	①→364
363	¿Su bebé fue envuelto en una toalla o tela inmediatamente después de nacido?	①. SÍ<br>⓪. NO<br>⑧. NO SABE/ NO SE ACUERDA	
364	¿Su bebé fue pesado inmediatamente después de nacido?<br>✎ En caso de gemelos, referirse al 2do gemelo	①. SÍ<br>⓪. NO<br>⑧. NO SABE/ NO SE ACUERDA	
365	¿Cuánto pesó su bebé?<br>✎ En caso de gemelos, referirse al 2do gemelo	[____].[__________]<br>PESO EN KILOGRAMOS<br>Ó<br>[____|____|____|____]<br>PESO EN GRAMOS	

CUESTIONARIO DE SALIDA A PACIENTES

No.	PREGUNTA/ ACCIÓN	CÓDIGO DE RESPUESTA	SALTAR A
	**SECCIÓN 3: TRABAJO DE PARTO Y EXPULSION**		
366	**Indicar la fuente de informacion para la cifra del peso**	①. PESO TOMADO DE LA CARTILLA DE SALUD Y/O EXPEDIENTE<br>②. PESO REPORTADO POR LA MADRE	
367	¿Usted amamantó a su bebé?<br>✑ En caso de gemelos, referirse al 2do gemelo	①. SÍ<br>⓪. NO	⓪→369
368	¿Usted amamantó a su bebé dentro de la primera hora después del parto?<br>✑ En caso de gemelos, referirse al 2do gemelo	①. SÍ<br>⓪. NO<br>⑧. NO SABE/ NO RECUERDA	
369	¿Usted, o alguien más, le dio algo de comer o beber al bebé dentro de la primera hora después del parto?<br>✑ En caso de gemelos, referirse al 2do gemelo<br>✑ Entrevistador(a): por favor contar líquidos aparte de leche materna.	①. SÍ<br>⓪. NO<br>⑧. NO SABE/ NO RECUERDA	
370	¿Cuánto tiempo después de nacido se bañó al bebé por primera vez?<br>✑ En caso de gemelos, referirse al 2do gemelo	①. DENTRO DE LA PRIMERA HORA<br>②. 1-5 HORAS<br>③. 6-24 HORAS<br>④. 2-3 DÍAS<br>⑤. DESPUÉS DE 3 DÍAS<br>⑧. NO SABE/ NO RECUERDA	
371	En la primera revisión que le hicieron después del parto, ¿el proveedor de salud buscó o le preguntó si estaba sangrando?	①. SÍ<br>⓪. NO<br>⑧. NO SABE/ NO RECUERDA	
372	¿Le hicieron una transfusión de sangre en cualquier momento del parto o de su estancia en el hospital?	①. SÍ<br>⓪. NO<br>⑧. NO SABE/ NO RECUERDA	
373	En la primera revisión que le hicieron después del parto, ¿el proveedor de salud le hizo una revisión perineal?<br>✑ Entrevistador(a): explique qué es revisión pèrineal	①. SÍ<br>⓪. NO<br>⑧. NO SABE/ NO SE ACUERDA	
374	En la primera revisión que le hicieron después del parto, ¿algún proveedor de salud revisó su panza para saber si su matriz estaba firme/ se endureció?	①. SÍ<br>⓪. NO<br>⑧. NO SABE/ NO SE ACUERDA	
375	En la primera revisión que le hicieron después del parto, ¿algún proveedor de salud le tomó la presión?	①. SÍ<br>⓪. NO<br>⑧. NO SABE/ NO SE ACUERDA	
376	En la primera revisión que le hicieron después del parto, ¿algún proveedor de salud le tomó la temperatura?	①. SÍ<br>⓪. NO<br>⑧. NO SABE/ NO SE ACUERDA	

ID PACIENTE [___|___] [___|___] [___|___][___]

CUESTIONARIO DE SALIDA A PACIENTES

No.	PREGUNTA/ ACCIÓN	CÓDIGO DE RESPUESTA	SALTAR A
	**SECCIÓN 3: TRABAJO DE PARTO Y EXPULSION**		
377	En algún momento durante su estancia en el hospital, ¿solicitó algún medicamento para aliviar el dolor?	①. SÍ<br>⓪. NO<br>⑧. NO SABE/ NO SE ACUERDA	
378	En algún momento durante su estancia en el hospital, ¿le dieron algún medicamento para aliviar el dolor?	①. SÍ<br>⓪. NO<br>⑧. NO SABE/ NO SE ACUERDA	
379	¿Aproximadamente cuánto tiempo pasó desde que ingresó al hospital y hasta que nació su bebé?	①. 1-3 HORAS<br>②. 3-6 HORAS<br>③. 6-12 HORAS<br>④. 12-14 HORAS<br>⑤. MÁS DE 24 HORAS<br>⑧. NO SABE/ NO RECUERDA	
380	¿Aproximadamente cuánto tiempo se quedó hospitalizada desde el momento en que nació su bebé?	①. 1-6 HORAS<br>②. 6-12 HORAS<br>③. 12-14 HORAS<br>④. 1-2 DÍAS<br>⑤. MÁS DE 3 DÍAS<br>⑧. NO SABE/ NO RECUERDA	
**381**	**HORA EN QUE FINALIZA LA ENTREVISTA**<br>✎ AGRADECER A LA ENTREVISTADA Y OFRECER VALE DE DESPENSA.<br>✎ COMPLETAR LA PREGUNTA 105	HORAS [___|___]<br>MINUTOS [___|___]	

382 NOTAS DE CAMPO DE LA ENTREVISTADORA

....

....

....

....

....

....

....

....

....

....

....

....

....
