# Supplementary material for: Measuring progress in maternal and newborn health care in Mexico: validating indicators of health system contact and quality of care
Source: BMC Pregnancy Childbirth. 2016 Aug 30;16(1):255. doi: 10.1186/s12884-016-1047-0 (PMC5006493; doi:10.1186/s12884-016-1047-0)
Supplement: Additional file 3: — Observation Checklist (English). (PDF 280 kb) [file 12884_2016_1047_MOESM3_ESM.pdf]

LABOR AND DELIVERY OBSERVATION CHECKLIST

Validating indicators of the quality of maternal health care

OBSERVER: *BEFORE BEGINNING OBSERVATION, INTRODUCE YOURSELF AND OBTAIN PERMISSION FROM BOTH THE SERVICE PROVIDER AND CLIENT. MAKE SURE THAT THE PROVIDER KNOWS THAT YOU ARE NOT THERE TO EVALUATE HIM OR HER, AND THAT YOU ARE NOT AN 'EXPERT' TO BE CONSULTED DURING THE SESSION.*

#	Question	Response codes	SKIP TO
SECTION 1: COVER SHEET INFORMATION			
101.	OBSERVER NAME	____________________________	
102.	OBSERVER CODE	|___|___|___|	
103.	FACILITY Name	____________________________	
104.	FACILITY CODE	|___|___|___|	
105.	CLIENT ID	|___|___||___|___||___|___||___|	
106.	DATE OF OBSERVATION	|___|___||___|___||___|___| D D M M Y Y	
107.	TIME OF DAY DELIVERY CONDUCTED	DAY........ 1<br>NIGHT........ 2	
108.	OUTCOME OF OBSERVATION<br><br>**COMPLETE AT END OF OBSERVATION**	COMPLETED........ 1<br>PARTIALLY COMPLETED........ 2<br>REFUSED........ 3<br>OTHER........ 8	END<br>END<br>END<br>END
109.	LANGUAGE(S) USED [BETWEEN PROVIDER AND CLIENT] TO CONDUCT OBSERVATION	ENGLISH........ 1<br>SPANISH / KISWAHILI........ 2<br>LOCAL LANGUAGE........ 3<br>(SPECIFY)<br>____________________________	

#	Question	Response codes	SKIP TO
110.	TIME OBSERVATION BEGINS: (USE THE 24-HOUR CLOCK)	HOURS |___|___| MINUTES |___|___|	

#	Question/Task	Response codes	SKIP TO
**SECTION 2: OBSERVATION OF INITIAL CLIENT ASSESSMENT**<br>*RECORD WHETHER THE PROVIDER CARRIED OUT THE FOLLOWING STEPS AND/OR EXAMINATIONS (SOME OF THE FOLLOWING STEPS MAY BE PERFORMED SIMULTANEOUSLY OR BY MORE THAN ONE PROVIDER)*			
200.	WAS THIS SECTION OBSERVED	YES ....1<br>NO...2	 300
201.	Record age of woman	|___|___|	
202.	Record parity of the woman prior to this delivery	|___|___|	
203.	Was woman referred from another facility?	YES ....1<br>NO...2<br>DK...8	
204.	Time of admission to labor ward (use 24 hour clock)	HOURS |___|___| MINUTES |___|___|	
205.	Encourages the woman to have a support person present throughout labor and birth	YES ....1<br>NO...2<br>DK...8	
206.	Checks woman's HIV status (checks chart or asks woman)?	YES ....1<br>NO...2<br>DK...8	
207.	Offers woman HIV test	YES ....1<br>NO...2<br>DK...8	
208.	Gives woman HIV test	YES ....1<br>NO...2<br>DK...8	

#	Question/Task	Response codes	SKIP TO
209.	Washes his/her hands with soap and water or uses antiseptic before any initial examination	YES .... 1<br>NO... 2<br>DK... 8	
210.	Explains procedures to woman (support person) before proceeding	YES .... 1<br>NO... 2<br>DK... 8	
211.	Takes temperature	YES .... 1<br>NO... 2<br>DK... 8	
212.	Takes pulse	YES .... 1<br>NO... 2<br>DK... 8	
213.	Takes blood pressure	YES .... 1<br>NO... 2<br>DK... 8	
214.	Takes urine sample to test for presence of protein	YES .... 1<br>NO... 2<br>DK... 8	
215.	Abdominal examination – Checks fetal heart rates with fetoscope/doppler/ultrasound	YES .... 1<br>NO... 2<br>DK... 8	
216.	Performs vaginal examination	YES .... 1<br>NO... 2<br>DK... 8	
217.	Wears high-level disinfected or sterile gloves for vaginal examination	YES .... 1<br>NO... 2<br>DK... 8	

#	Question/Task	Response codes	SKIP TO
**SECTION 3: OBSERVATION OF 1st STAGE OF LABOUR**<br><br>*RECORD WHETHER THE PROVIDER CARRIED OUT THE FOLLOWING STEPS AND/OR EXAMINATIONS (SOME OF THE FOLLOWING STEPS MAY BE PERFORMED SIMULTANEOUSLY OR BY MORE THAN ONE PROVIDER)*			
300.	WAS THIS SECTION OBSERVED	YES ....1<br>NO..2	 400
301.	Induces labor with uterotonic	YES ....1<br>NO..2<br>DK..8	 303<br>303
302.	Route of administration of utertonic	IV..1<br>IM...2	
303.	Augments labor with uterotonic	YES ....1<br>NO..2<br>DK..8	 305<br>305
304.	Route of administration of utertonic	IV..1<br>IM...2	
305.	Performs artificial rupture of the membranes	YES ....1<br>NO..2<br>DK..8	
306.	At least once, encourages/assists the woman to ambulate during labor	YES ....1<br>NO..2<br>DK..8	
307.	At least once, encourages/assists the woman to assume different positions during labor	YES ....1<br>NO..2<br>DK..8	
308.	At least once, encourages/assists the woman to drink liquids or eat	YES ....1<br>NO..2<br>DK..8	
309.	Encourages the woman to have a support person present throughout labor and birth	YES ....1<br>NO..2<br>DK..8	

#	Question/Task	Response codes	SKIP TO
310.	A support person is present at some point during labor	YES ....1<br>NO...2<br>DK...8	
311.	Washes his/her hands with soap and water or uses antiseptic before examination of the woman	YES ....1<br>NO...2<br>DK...8	
312.	Wears high-level disinfected or sterile gloves	YES ....1<br>NO...2<br>DK...8	
313.	Who was the main provider(s) who assisted with the woman's labor?<br><br>LIST ALL PROVIDERS	DOCTOR (OBGYN).................1<br>MEDICAL RESIDENT ....2<br>MEDICAL INTERN.................3<br>NURSE..................................4<br>MIDWIFE...............................5<br>CLINCAL OFFICER.................6<br>SUPPORT STAFF/AIDE..........7<br>OTHER ....8<br>(SPECIFY)<br><br>___________________________	
314.	Did other provider(s) assist with the woman's labor?<br><br>LIST ALL PROVIDERS	DOCTOR (OBGYN).................1<br>MEDICAL RESIDENT ....2<br>MEDICAL INTERN.................3<br>NURSE..................................4<br>MIDWIFE...............................5<br>CLINCAL OFFICER.................6<br>SUPPORT STAFF/AIDE..........7<br>OTHER ....8<br>(SPECIFY)<br><br>___________________________	
315.	How many providers in total assisted with the woman's labor?	|___|___|	

#	Question/Task	Response codes	SKIP TO
**SECTION 4: CONTINUOUS OBSERVATION OF SECOND & THIRD STAGE OF LABOR**<br><br>*RECORD WHETHER THE PROVIDER CARRIED OUT THE FOLLOWING STEPS AND/OR EXAMINATIONS (SOME OF THE FOLLOWING STEPS MAY BE PERFORMED SIMULTANEOUSLY OR BY MORE THAN ONE PROVIDER)*			
400.	WAS THIS SECTION OBSERVED	YES ....1<br>NO..2	 500
401.	Was an episiotomy done?	YES ....1<br>NO..2<br>DK..8	
402.	OBSERVER: Note time of the delivery of the baby<br>(use 24 hour clock)	HOURS |___|___| <br>MINUTES |___|___|	
403.	Second baby present	YES (twin) ....1<br>NO (singleton).......................2	
404.	Birthweight (in grams); enter 9999 if not available	|___|___||___|___|	
405.	Uterotonic administered	YES ....1<br>NO..2<br>DK..8	 411<br>411
406.	OBSERVER: Note time uterotonic given (use 24 hour clock)	HOURS |___|___| <br>MINUTES |___|___|	

#	Question/Task	Response codes	SKIP TO
407.	Timing of administration of uterotonic:<br>a. at delivery of the anterior shoulder<br>b. within 1 minute of delivery of baby<br>c. within 3 minutes of delivery of baby<br>d. more than 3 minutes of delivery of baby AND BEFORE delivery of the placenta<br>e. more than 3 minutes of delivery of baby AND AFTER delivery of the placenta	ANT SHLDR .... 1<br>1 MIN AFTER DELIVERY .... 2<br>3 MINS AFTER DELIVERY ..... 3<br>+3 BEFORE PLACENTA......... 4<br>+3 AFTER PLACENTA............ 5<br>DK... 8	
408.	Which uterotonic given:<br>a. Oxytocin<br>b. Ergometrine<br>c. Syntometrine<br>d. Prostaglandins	OXYTOCIN.............................. 1<br>ERGOMETRINE .... 2<br>SYNTOMETRINE..................... 3<br>PROSTAGLANDIN.................. 4<br>DK... 8	
409.	Dose of uterotonic given and type of units of medication<br>(e.g. IU, mg)<br><br>(IF NECESSARY, ASK/CHECK AFTERWARDS)	D) DOSE |___|___| <br>U) UNITS<br>IU .... 1<br>mg.. 2<br>ml... 3<br>mcg...................................... 4	
410.	Route uterotonic given	IM... 1<br>IV push (bolus)...................... 2<br>IV drip .... 3<br>IV drip plus IM...................... 4<br>Oral .... 5<br>Vaginal .... 6<br>Sublingual .... 7<br>Rectal 8	
411.	OBSERVER: Note time the cord was clamped<br>(use 24-hour clock)	HOURS |___|___| <br>MINUTES |___|___|	

#	Question/Task	Response codes	SKIP TO
412.	Applies traction to cord while applying suprapubic counter traction	YES ....1<br>NO...2<br>DK...8	
413.	Performs uterine massage immediately following delivery	YES ....1<br>NO...2<br>DK...8	
414.	Assesses completeness of the placenta and membranes	YES ....1<br>NO...2<br>DK...8	
415.	Assesses for perineal and vaginal lacerations	YES ....1<br>NO...2<br>DK...8	
416.	Did more than one health worker assist with the birth?	YES ....1<br>NO...2	
417.	Who was the main provider(s) who assisted with the delivery?<br><br>LIST ALL PROVIDERS	DOCTOR (OBGYN)..................1<br>MEDICAL RESIDENT ....2<br>MEDICAL INTERN..................3<br>NURSE...................................4<br>MIDWIFE................................5<br>CLINCAL OFFICER.................6<br>SUPPORT STAFF/AIDE..........7<br>OTHER ....8<br>(SPECIFY)<br>________________________	
418.	Did other provider(s) assist with the delivery?<br><br>LIST ALL PROVIDERS	DOCTOR (OBGYN)..................1<br>MEDICAL RESIDENT ....2<br>MEDICAL INTERN..................3<br>NURSE...................................4<br>MIDWIFE................................5<br>CLINCAL OFFICER.................6<br>SUPPORT STAFF/AIDE..........7<br>OTHER ....8<br>(SPECIFY)<br>________________________	

#	Question/Task	Response codes	SKIP TO
419.	How many providers in total assisted with the woman's delivery?	|___|___|	
420.	What was the position of the mother at birth?	ON BACK ....1<br>(LITHOTOMY)<br>UPRIGHT HALF-SITTING........2<br>LEFT LATERAL.......................3<br>ON HANDS AND KNEES ....4<br>SQUATTING ....5<br>OTHER ....8<br>(SPECIFY)<br>___________________________	

#	Question/Task	Response codes	SKIP TO
**SECTION 5: OBSERVATION OF IMMEDIATE NEWBORN, POSTNATAL AND POSTPARTUM CARE**<br><br>*RECORD WHETHER THE PROVIDER CARRIED OUT THE FOLLOWING STEPS AND/OR EXAMINATIONS (SOME OF THE FOLLOWING STEPS MAY BE PERFORMED SIMULTANEOUSLY OR BY MORE THAN ONE PROVIDER)*			
500.	WAS THIS SECTION OBSERVED	YES ....1<br>NO..2	 600
501.	A support person (companion) for mother is present during the birth	YES ....1<br>NO..2<br>DK..8	
502.	Immediately dries baby with towel /cloth	YES ....1<br>NO..2<br>DK..8	
503.	Is the baby breathing /crying?	YES ....1<br>NO..2	 514
504.	Baby given to mother immediately, within a few minutes, after birth?	YES ....1<br>NO..2<br>DK..8	
505.	Places newborn on the mother's abdomen "skin to skin"	YES ....1<br>NO..2<br>DK..8	 507
506.	Covers baby with dry towel	YES ....1<br>NO..2<br>DK..8	
507.	If not placed skin to skin, wraps baby in dry towel	YES ....1<br>NO..2<br>DK..8	
508.	Ties or clamps cord when pulsations stop or by 2-3 minutes after birth (not immediately after birth)	YES ....1<br>NO..2<br>DK..8	

#	Question/Task	Response codes	SKIP TO
509.	Cuts cord, using clean blade or clean scissors	YES .... 1<br>NO.. 2<br>DK.. 8	
510.	Breastfeeding initiated within the first hour after birth	YES .... 1<br>NO.. 2<br>DK.. 8	
511.	Was anything besides breastmilk given to the baby to drink within the first hour after birth?	YES .... 1<br>NO.. 2<br>DK.. 8	
512.	Baby bathed within the first hour after birth	YES .... 1<br>NO.. 2<br>DK.. 8	
513.	Baby kept skin to skin with mother for first hour after birth	YES .... 1<br>NO.. 2<br>DK.. 8	
514.	Takes mother's vital signs 15 minutes after birth	YES .... 1<br>NO.. 2<br>DK.. 8	
515.	Palpates uterus 15 minutes after delivery of placenta	YES .... 1<br>NO.. 2<br>DK.. 8	
516.	In first physical examination after delivery – *during or after the 4th stage of labor* – did health provider look for or ask about bleeding?	YES .... 1<br>NO.. 2<br>DK.. 8	
517.	In first physical examination after delivery – *during or after the 4th stage of labor* – did health provider examine the perineum?	YES .... 1<br>NO.. 2<br>DK.. 8	
518.	In first physical examination after delivery – *during or after the 4th stage of labor* – did health provider take the woman's temperature?	YES .... 1<br>NO.. 2<br>DK.. 8	

#	Question/Task	Response codes	SKIP TO
519.	In first physical examination after delivery – *during or after the 4th stage of labor* – did health provider take the woman's blood pressure?	YES .... 1<br>NO.. 2<br>DK.. 8	
520.	In first physical examination after delivery – *during or after the 4th stage of labor* – did health provider check for involution?	YES .... 1<br>NO.. 2<br>DK.. 8	
521.	Time of first physical examination of the mother after delivery<br>(use 24 hour clock)	HOURS |___|___| <br>MINUTES |___|___|	
522.	Time of admission to post-labor ward<br>(use 24 hour clock)	HOURS |___|___| <br>MINUTES |___|___|	

#	Question/Task	Response codes	SKIP TO
**SECTION 6: REVIEW OF L&D OBSERVATION**<br><br>IF NECESSARY, CHECK MEDICAL RECORDS/ ASK PROVIDER AFTER OBSERVATION			
600.	Did the provider complete maternity register/birth log	YES......................................1<br>NO..2<br>DK..8	
601.	Did the provider use a partograph	YES......................................1<br>NO..2<br>DK..8	603
602.	Partograph not used because woman arrived in second stage of labor	YES......................................1<br>NO..2<br>DK..8	
603.	OBSERVER: did you see provider fill out partograph after delivery<br>(with information that should be entered during labor)	YES......................................1<br>NO..2<br>DK..8	
604.	Record the condition/outcome for the mother<br>a. Goes to recuperation ward<br>b. Referred to specialist in same facility<br>c. Goes to surgery in same facility<br>d. Referred to other facility<br>e. Death of mother	RECUP WARD ....1<br>REFER, SAME FACILITY.........2<br>SURGERY..............................3<br>REFER, OTHER FACILITY.......4<br>DEATH..................................5	
605.	Record the condition/outcome for the baby<br>a. Goes to normal nursery<br>b. Referred to specialist in same facility<br>c. Referred to other facility<br>d. Goes to ward with mother<br>e. Newborn death<br>f. Fresh stillbirth<br>g. Macerated stillbirth	NURSERY..............................1<br>REFER, SAME FACILITY.........2<br>REFER, OTHER FACILITY.......3<br>WARD WITH MOTHER ....4<br>NEWBORN DEATH.................5<br>FRESH STILLBIRTH ....6<br>MACERATED STILLBIRTH 7	

#	Question/Task	Response codes	SKIP TO
606.	Record the type of delivery	VAGINAL ....1<br>ASSISTED, FORCEPS ....2<br>ASSISTED, VENTOUSE ....3<br>CESAREAN..............................4<br>DK...8	611<br>611<br>611<br><br>611
607.	Was cesarean operation performed before woman went into labor or after labor had already started?	BEFORE LABOR STARTED ....1<br>AFTER LABOR STARTED ....2<br>DON'T KNOW.........................8	
608.	What was the indication for the cesarean operation?	Excessive bleeding ....1<br>Obstructed labor...................2<br>Prolonged labor.....................3<br>Uterine rupture ....4<br>Malpresentation ....5<br>Fetal distress ....6<br>OTHER ....7<br>(SPECIFY)<br>______________________<br>No medical reason................8<br>Don't know 9	
609.	When was the decision for the cesarean taken?	BEFORE LABOR STARTED ....1<br>AFTER LABOR STARTED ....2<br>DON'T KNOW.........................8	
610.	Who decided cesarean would be done?	DOCTOR/PROVIDER ....1<br>WOMAN ....2<br>DON'T KNOW.........................8	

#	Question/Task	Response codes	SKIP TO
611.	Record if mother had any of the following complications:<br><br>a. Preeclampsia/Elampsia<br>b. Hemorrhage<br>c. Obstructed labor<br>d. Prolonged labor<br>e. Uterine rupture<br>f. Puerperal infection	PE/E......................................1<br>HEMORRHAGE ....2<br>OBSTRUCTED LABOR............3<br>PROLONGED LABOR ....4<br>UTERINE RUPTURE ....5<br>SEPSIS ....6<br>OTHER ....7<br>(SPECIFY)<br>_________________________<br>NO COMPLICATIONS.............8<br>DK...9	
612.	At what stage of labor and delivery did the complication occur?	BEFORE DELIVERY................1<br>AT DELIVERY ....2<br>POST PARTUM.......................3<br>DK...8	
613.	Were any blood products given to the women due to excessive blood loss during labor, delivery or immediate postpartum?	YES.......................................1<br>NO...2<br>DK...8	
614.	Did the woman ask for any pain relief medication during labor, delivery or immediate postpartum?	YES.......................................1<br>NO...2<br>DK...8	
615.	Was the woman given any pain relief medication during labor, delivery or immediate postpartum?	YES.......................................1<br>NO...2<br>DK...8	
616.	Was the woman given any antibiotics during labor, delivery or immediate postpartum?	YES.......................................1<br>NO...2<br>DK...8	
617.	Time L&D observation ended<br>(use 24-hour clock)	HOURS |___|___| <br>MINUTES |___|___|	

#	Question/Task	Response codes	SKIP TO
618.	OBSERVER WRITTEN NOTES.<br>In particular, comment about the following:<br><br>*Was mother treated respectfully? Informed of procedures to herself and her baby?*<br>*Was the situation chaotic or calm and ordered?*<br>*Were there any major delays in needed treatment? If so, for what procedures (mother or baby) and why? Were multiple health workers involved? Who?*<br>*If baby died, was mother counseled?*<br>*If unable to complete the observation, why?*<br><br>____________________		
619.	**END**		
