# Supplementary material for: Measuring progress in maternal and newborn health care in Mexico: validating indicators of health system contact and quality of care
Source: BMC Pregnancy Childbirth. 2016 Aug 30;16(1):255. doi: 10.1186/s12884-016-1047-0 (PMC5006493; doi:10.1186/s12884-016-1047-0)
Supplement: Additional file 4: — Observation Checklist (Spanish). (PDF 1175 kb) [file 12884_2016_1047_MOESM4_ESM.pdf]

SALUD
HOSPITAL GENERAL de MÉXICO
Population Council

ID PACIENTE [___|___] [___|___] [___|___][___]

ANEXO I **LISTA DE OBSERVACIONES DE TRABAJO DE PARTO Y EXPULSIÓN**

△ **OBSERVADOR(A)**: PRESÉNTESE Y OBTENGA PERMISO DEL PROVEEDOR DE SALUD Y DE LA PACIENTE ANTES DE INICIAR LA OBSERVACIÓN. ASEGÚRESE DE QUE EL PROVEEDOR SEPA QUE NO LO(A) ESTA EVALUANDO Y QUE USTED NO ES UN "EXPERTO" A QUIÉN PUEDA CONSULTAR DURANTE LA SESION

No.	PREGUNTA	CÓDIGO DE RESPUESTA	PATRÓN DE SALTO
	**SECCIÓN 1: INFORMACIÓN DE LA CARÁTULA**		
101	NOMBRE DEL (DE LA) OBSERVADOR(A)		
102	CÓDIGO DEL (DE LA) OBSERVADOR(A)	[__ __ __]	
103	NOMBRE DEL HOSPITAL		
104	CÓDIGO DEL HOSPITAL	[__ __ __]	
105	IDENTIFICACIÓN DE LA PACIENTE	[__ __ __] [__ __ __] [__ __ __]	
106	FECHA DE LA OBSERVACIÓN	[__ __] [__ __] [__ __] DD MM AA	
107	HORA DEL DÍA EN LA CUAL OCURRIÓ EL PARTO	①. MATUTINO<br>②. VESPERTINO<br>③. NOCTURNO	
108	RESULTADO DE LA OBSERVACIÓN<br>✎ COMPLETAR UNA VEZ FINALIZADA LA OBSERVACIÓN	①.COMPLETADA<br>②. PARCIALMENTE COMPLETADA<br>③. RECHAZADA<br>⑧. OTRA	→FIN<br>→FIN<br>→FIN<br>→FIN
109	HORA EN LA QUE INICIA LA OBSERVACIÓN<br>✎ UTILICE EL RELOJ DE 24 HORAS	HORAS [___|___]<br>MINUTOS [___|___]	

TdP: Trabajo de parto

ANEXO I **LISTA DE OBSERVACIONES DE TRABAJO DE PARTO Y EXPULSIÓN**

No.	PREGUNTA/ ACCIÓN	CÓDIGO DE RESPUESTA	SALTAR A
△	**SECCIÓN 2: OBSERVACIÓN DE LA EVALUACION INICIAL DE LA PACIENTE**<br>REGISTRE SI EL PROVEEDOR DE SALUD REALIZÓ LAS SIGUIENTES ACCIONES Y/O EXÁMENES (ALGUNOS PUEDEN REALIZARSE DE FORMA SIMULTÁNEA O POR MÁS DE UN PROVEEDOR)		
200	¿SE OBSERVÓ ESTA SECCIÓN?	①.SÍ ②.NO	②→300
201	Registre la edad de la mujer	[___|___]	
202	Registre la paridad de la mujer, **previo** a este parto	[___|___]	
203	¿La mujer ha sido referida de otra institución de salud?	①.SÍ ②.NO ⑧.NO SABE	
204	HORA DE ADMISIÓN A LA SALA DE PARTO<br>✎ Utilice el reloj de 24 horas	HORAS [___|___]<br>MINUTOS [___|___]	
205	¿Se alienta a la mujer para que tenga un acompañante presente a lo largo del trabajo de parto y expulsión?	①.SÍ ②.NO ⑧.NO SABE	
206	¿Se revisa si la mujer tiene VIH (revisa el expediente clínico o se le pregunta a la mujer)?	①.SÍ ②.NO ⑧.NO SABE	
207	¿Se ofrece a la mujer una prueba de VIH?	①.SÍ ②.NO ⑧.NO SABE	
208	¿Se realiza una prueba de VIH?	①.SÍ ②.NO ⑧.NO SABE	
209	¿Se lava las manos con agua y jabón o utiliza un antiséptico antes de cada exploración a la mujer?	①.SÍ ②.NO ⑧.NO SABE	
210	¿Se explican los procedimientos a la mujer (acompañante) antes de realizarlos?	①.SÍ ②.NO ⑧.NO SABE	
211	¿Se toma la temperatura?	①.SÍ ②.NO ⑧.NO SABE	
212	¿Se toma el puslo?	①.SÍ ②.NO ⑧.NO SABE	
213	¿Se toma la tensión arterial?	①.SÍ ②.NO ⑧.NO SABE	
214	¿Se toma muestra de orina para evaluar presencia de proteínas?	①.SÍ ②.NO ⑧.NO SABE	
215	Exploración abdominal –<br>¿Se toma la frecuencia cardiaca fetal con fetoscopio/ doppler/ ultrasonido?	①.SÍ ②.NO ⑧.NO SABE	
216	¿Se realiza tacto vaginal?	①.SÍ ②.NO ⑧.NO SABE	②→300
217	¿Utiliza guantes desinfectados o estériles para realizar el tacto vaginal?	①.SÍ ②.NO ⑧.NO SABE	

ANEXO I **LISTA DE OBSERVACIONES DE TRABAJO DE PARTO Y EXPULSIÓN**

No.	PREGUNTA/ ACCIÓN	CÓDIGO DE RESPUESTA	SALTAR A
△	**SECCIÓN 3: OBSERVACIÓN DE LA PRIMERA ETAPA DEL TRABAJO DE PARTO**<br>REGISTRE SI EL PROVEEDOR DE SALUD REALIZÓ LAS SIGUIENTES ACCIONES Y/O EXÁMENES (ALGUNOS PUEDEN REALIZARSE DE FORMA SIMULTÁNEA O POR MÁS DE UN PROVEEDOR)		
300	¿SE OBSERVÓ ESTA SECCIÓN?	①. SÍ ②. NO	②→400
301	Se induce el trabajo de parto con un uterotónico?	①. SÍ ②. NO ⑧. NO SABE	②→303<br>⑧→303
302	Vía de administración del uterotónico	①. IV ②. IM	
303	¿El trabajo de parto se lleva a cabo con un uterotónico?	①. SÍ ②. NO ⑧. NO SABE	②→305<br>⑧→305
304	Vía de administración del uterotónico	①. IV ②. IM	
305	¿Se lleva a cabo la rotura artificial de membranas? (Amniotomía)	①.SÍ ②.NO ⑧.NO SABE	
306	Alienta / ayuda a la mujer a caminar durante el trabajo de parto, al menos en una ocasión	①.SÍ ②.NO ⑧.NO SABE	
307	Alienta / ayuda a la mujer a adoptar diferentes posiciones durante el trabajo de parto, al menos en una ocasión	①.SÍ ②.NO ⑧.NO SABE	
308	Alienta / ayuda a la mujer a beber líquidos o comer, al menos en una ocasión	①.SÍ ②.NO ⑧.NO SABE	
309	Alienta a la mujer para que tenga un acompañante presente a lo largo del trabajo de parto y expulsión	①.SÍ ②.NO ⑧.NO SABE	
310	Está presente un acompañante en algún momento del trabajo de parto	①.SÍ ②.NO ⑧.NO SABE	
311	Se lava las manos con agua y jabón o utiliza un antiséptico antes de cada exploración a la mujer	①.SÍ ②. NO ⑧. NO SABE	
312	Utiliza guantes desinfectados o estériles	①.SÍ ②.NO ⑧.NO SABE	
313	¿Cuál fue el PRINCIPAL PROVEEDOR DE SALUD que atendió EL TRABAJO DE PARTO?<br>✎ Por favor especificar solo la persona principal que atendió el trabajo de parto.	①. MÉDICO<br>②. MÉDICO GINECÓLOGO<br>③. MÉDICO RESIDENTE<br>④. MÉDICO INTERNO<br>⑤. ESTUDIANTE DE MEDICINA<br>⑥. ENFERMERA<br>⑦. PASANTE DE ENFERMERÍA<br>⑧. ESTUDIANTE DE ENFERMERÍA<br>⑨. PARTERA<br>⑩. OTRO<br>ESPECIFICAR:	
314	¿Hubieron OTROS PROVEEDORES DE SALUD que atendieron EL TRABAJO DE PARTO?<br>✎ Enliste todos los proveedores que atendieron el trabajo de parto aparte de la persona principal.	①. MÉDICO<br>②. MÉDICO GINECÓLOGO<br>③. MÉDICO RESIDENTE<br>④. MÉDICO INTERNO<br>⑤. ESTUDIANTE DE MEDICINA<br>⑥. ENFERMERA<br>⑦. PASANTE DE ENFERMERÍA<br>⑧. ESTUDIANTE DE ENFERMERÍA<br>⑨. PARTERA<br>⑩. OTRO<br>ESPECIFICAR:	
315	¿Cuántos proveedores, en total, atendieron el parto?	[___|___]	

No.	PREGUNTA/ ACCIÓN	CÓDIGO DE RESPUESTA	SALTAR A
△	**SECCIÓN 4: OBSERVACIÓN CONTINUA DE LA SEGUNDA Y TERCERA ETAPA DEL PARTO**<br>REGISTRE SI EL PROVEEDOR DE SALUD REALIZÓ LAS SIGUIENTES ACCIONES Y/O EXAMENES (ALGUNOS PUEDEN REALIZARSE DE FORMA SIMULTÁNEA O POR MÁS DE UN PROVEEDOR)		
400	¿SE OBSERVÓ ESTA SECCIÓN?	①. SÍ ②. NO	②→500
401	¿Se realizó una episiotomía?	①. SÍ ②. NO ⑧. NO SABE	
402	OBSERVADOR(A): Anote la hora del parto<br>✒ Utilice el reloj de 24 horas	HORAS [___|___]<br>MINUTOS [___|___]	
403	Presencia de segundo bebé	①. SÍ (gemelar)<br>②. NO (producto único)	
404	Peso al nacimiento (en gramos); registre 9999 si no está disponible	[___|___] [___|___]	
405	Uterotónico administrado	①.SÍ ②.NO ⑧.NO SABE	②→411<br>⑧→411
406	OBSERVADOR(A): Anote la hora de administración del uterotónico<br>✒ Utilice el reloj de 24 horas	HORAS [___|___]<br>MINUTOS [___|___]	
407	Anotar el momento en que se administró el uterotónico:<br>a. Al nacimiento, hombro anterior<br>b. Dentro del primer minuto posterior al nacimiento del bebé<br>c. Dentro de los primeros 3 minutos posteriores al nacimiento del bebé<br>d. Más de 3 minutos después de nacido el bebé y ANTES de la expulsión de la placenta<br>e. Más de 3 minutos después de nacido el bebé Y DESPUES de la expulsión de la placenta	①. Hombro anterior<br>②. 1 MIN POSTERIOR NAC.<br>③. 3 MINS POSTERIOR NAC.<br>④. +3 ANTES DE PLACENTA<br>⑤. +3 DESPUÉS DE PLACENTA<br>⑧. NO SABE	
408	¿Que uterotónico se administró?:<br>a. Oxitocina<br>b. Ergonovina<br>c. Sintometrina<br>d. Prostaglandina	①. OXITOCINA<br>②. ERGONOVINA<br>③. SINTOMETRINA<br>④. PROSTAGLANDINAS<br>⑧. NO SABE	
409	DOSIS Y UNIDADES (e.j. UI, mg) del uterotónico administrado<br>✒ En caso necesario, preguntar/revisar después	DOSIS [___|___]<br>UNIDADES<br>①. UI<br>②. mg<br>③. ml<br>④. mcg	
410	Vía de administración del uterotónico	①. IM<br>②. IV EN BOLO<br>③. IV POR GOTEO<br>④. IV POR GOTEO MÁS IM<br>⑤. ORAL<br>⑥. VAGINAL<br>⑦. SUBLINGUAL<br>⑧. RECTAL	

ID PACIENTE [___|___] [___|___] [___|___][___]

ANEXO I

LISTA DE OBSERVACIONES DE TRABAJO DE PARTO Y EXPULSIÓN

No.	PREGUNTA/ ACCIÓN	CÓDIGO DE RESPUESTA	SALTAR A
△	**SECCIÓN 4: OBSERVACIÓN CONTINUA DE LA SEGUNDA Y TERCERA ETAPA DEL PARTO**<br>REGISTRE SI EL PROVEEDOR DE SALUD REALIZÓ LAS SIGUIENTES ACCIONES Y/O EXÁMENES (ALGUNOS PUEDEN REALIZARSE DE FORMA SIMULTÁNEA O POR MÁS DE UN PROVEEDOR)		
411	OBSERVADOR(A): Anote la hora en que se pinzó el cordón umbilical<br>✏ Utilice el reloj de 24 horas	HORAS [___|___]<br>MINUTOS [___|___]	
412	Aplica tracción al cordón umbilical mientras realiza contratracción (presión) suprapúbica	①.SÍ ②.NO ⑧.NO SABE	
413	Realiza masaje uterino inmediatamente después del parto	①.SÍ ②.NO ⑧.NO SABE	
414	Evalúa que la placenta y membranas estén completas	①.SÍ ②.NO ⑧.NO SABE	
415	Evalúa la presencia de laceraciones perineales y vaginales	①.SÍ ②.NO ⑧.NO SABE	
416	¿Participó más de un proveedor de salud en la atención del parto?	①.SÍ ②.NO	
417	¿Cuál fue el PRINCIPAL PROVEEDOR DE SALUD que atendió EL PARTO?<br>✏ Por favor especificar solo la persona principal que atendió el parto.	①. MÉDICO<br>②. MÉDICO GINECÓLOGO<br>③. MÉDICO RESIDENTE<br>④. MÉDICO INTERNO<br>⑤. ESTUDIANTE DE MEDICINA<br>⑥. ENFERMERA<br>⑦. PASANTE DE ENFERMERÍA<br>⑧. ESTUDIANTE DE ENFERMERÍA<br>⑨. PARTERA<br>⑩. OTRO<br>ESPECIFICAR:	
418	¿Hubieron otros PROVEEDORES DE SALUD que atendieron EL PARTO?<br>✏ Enliste todos los proveedores que atendieron el parto aparte de la persona principal.	①. MÉDICO<br>②. MÉDICO GINECÓLOGO<br>③. MÉDICO RESIDENTE<br>④. MÉDICO INTERNO<br>⑤. ESTUDIANTE DE MEDICINA<br>⑥. ENFERMERA<br>⑦. PASANTE DE ENFERMERÍA<br>⑧. ESTUDIANTE DE ENFERMERÍA<br>⑨. PARTERA<br>⑩. OTRO<br>ESPECIFICAR:	
419	¿Quién fue el PRINCIPAL PROVEEDOR DE SALUD que la atendió CUANDO NACIÓ SU BEBÉ (QUIÉN RECIBIÓ AL BEBÉ)?<br>✏ Esperar respuesta espontánea de la mujer.<br>✏ Por favor especificar solo la persona principal que la atendió cuando su bebé nació (quién recibió al bebé).	①. MÉDICO<br>②. MÉDICO GINECÓLOGO<br>③. MÉDICO RESIDENTE<br>④. MÉDICO INTERNO<br>⑤. ESTUDIANTE DE MEDICINA<br>⑥. ENFERMERA<br>⑦. PASANTE DE ENFERMERÍA<br>⑧. ESTUDIANTE DE ENFERMERÍA<br>⑨. PARTERA<br>⑩. OTRO<br>ESPECIFICAR:	

ANEXO I **LISTA DE OBSERVACIONES DE TRABAJO DE PARTO Y EXPULSIÓN**

No.	PREGUNTA/ ACCIÓN	CÓDIGO DE RESPUESTA	SALTAR A
△	**SECCIÓN 4: OBSERVACIÓN CONTINUA DE LA SEGUNDA Y TERCERA ETAPA DEL PARTO**<br>REGISTRE SI EL PROVEEDOR DE SALUD REALIZÓ LAS SIGUIENTES ACCIONES Y/O EXÁMENES (ALGUNOS PUEDEN REALIZARSE DE FORMA SIMULTÁNEA O POR MÁS DE UN PROVEEDOR)		
420	¿Cuántos proveedores, en total, atendieron el parto?	[___|___]	
421	¿En qué posición parió la mujer?	①. BOCA ARRIBA (LITOTOMIA)<br>②. SEMI-SENTADA<br>③. DECUBITO LATERAL IZQ.<br>④. SOBRE MANOS Y RODILLAS<br>⑤. EN CUCLILLAS<br>⑧. OTRO<br>ESPECIFICAR:	
△	**SECCIÓN 5: OBSERVACIÓN DE CUIDADOS NEONATALES INMEDIATOS, POSTNATALES Y DEL POSPARTO**<br>REGISTRE SI EL PROVEEDOR DE SALUD REALIZÓ LAS SIGUIENTES ACCIONES Y/O EXÁMENES (ALGUNOS PUEDEN REALIZARSE DE FORMA SIMULTÁNEA O POR MÁS DE UN PROVEEDOR)		
500	¿SE OBSERVO ESTA SECCIÓN?	①.SÍ ②.NO	②→600
501	Se encuentra presente un acompañante de la mujer durante el parto	①.SÍ ②.NO ⑧.NO SABE	
502	Se seca al bebé de forma inmediata con una toalla / tela	①.SÍ ②.NO ⑧.NO SABE	
503	¿El bebé está respirando / llorando?	①.SÍ ②.NO	②→514
504	¿Se entrega el bebé a la madre de forma inmediata al parto o pocos minutos después?	①.SÍ ②.NO ⑧.NO SABE	
505	¿Se coloca al recién nacido en el abdomen de la mujer, haciendo contacto "piel con piel"?	①.SÍ ②.NO ⑧.NO SABE	②→507
506	¿Se cubre al bebé con una toalla (o campo) seca?	①.SÍ ②.NO ⑧.NO SABE	
507	Si no es colocado "piel con piel", ¿se envuelve al bebé con una toalla (o campo) seca?	①.SÍ ②.NO ⑧.NO SABE	
508	¿Se amarra o pinza el cordón umbilical cuando las pulsaciones se detienen o a los 2-3 minutos después del parto (no de forma inmediata posterior a la expulsión)?	①.SÍ ②.NO ⑧.NO SABE	
509	¿Se corta el cordón umbilical con una navaja o tijeras limpias?	①.SÍ ②.NO ⑧.NO SABE	
510	¿Se inicia la lactancia materna dentro de la primera hora después del nacimiento?	①.SÍ ②.NO ⑧.NO SABE	
511	Además de leche materna, ¿se le dio al bebé algún líquido para tomar dentro de la primera hora después del nacimiento?<br>✎ Observador(a): por favor contar líquidos aparte de leche materna.	①.SÍ ②.NO ⑧.NO SABE	
512	¿Se bañó al bebé dentro de la primera hora después de nacido?	①.SÍ ②.NO ⑧.NO SABE	
513	¿Se mantuvo al bebé en contacto "piel con piel" con la madre durante la primera hora después de nacido?	①.SÍ ②.NO ⑧.NO SABE	

LISTA DE OBSERVACIONES DE TRABAJO DE PARTO Y EXPULSIÓN

No.	PREGUNTA/ ACCIÓN	CÓDIGO DE RESPUESTA	SALTAR A
△	**SECCIÓN 5: OBSERVACIÓN DE CUIDADOS NEONATALES INMEDIATOS, POSTNATALES Y DEL POSPARTO**<br>REGISTRE SI EL PROVEEDOR DE SALUD REALIZÓ LAS SIGUIENTES ACCIONES Y/O EXÁMENES (ALGUNOS PUEDEN REALIZARSE DE FORMA SIMULTÁNEA O POR MÁS DE UN PROVEEDOR)		
514	¿Se toman los signos vitales de la madre a los 15 minutos después del parto?	①.SÍ ②.NO ⑧.NO SABE	
515	¿Se realiza una palpación del útero a los 15 minutos después de la expulsión de la placenta?	①.SÍ ②.NO ⑧.NO SABE	
516	Durante la primera exploración después del parto – durante o después de la 4ta etapa del parto- ¿el proveedor busco o interrogó a la mujer sobre presencia de sangrado?	①.SÍ ②.NO ⑧.NO SABE	
517	Durante la primera exploración después del parto – durante o después de la 4ta etapa del parto- ¿el proveedor examinó el periné?	①.SÍ ②.NO ⑧.NO SABE	
518	Durante la primera exploración después del parto - durante o después de la 4ta etapa del parto- ¿el proveedor le tomó la temperatura a la mujer?	①.SÍ ②.NO ⑧.NO SABE	
519	Durante la primera exploración después del parto – durante o después de la 4ta etapa del parto- ¿el proveedor le tomó la presión a la mujer?	①.SÍ ②.NO ⑧.NO SABE	
520	Durante la primera exploración después del parto – durante o después de la 4ta etapa del parto- ¿el proveedor revisó la involución uterina?	①.SÍ ②.NO ⑧.NO SABE	
521	**Hora en la que se realiza la primera exploración física a la mujer después del parto**<br>✏ Utilice el reloj de 24 horas	HORAS [___|___]<br>MINUTOS [___|___]	
522	**Hora de admisión a la sala de recuperación**<br>✏ Utilice el reloj de 24 horas	HORAS [___|___]<br>MINUTOS [___|___]	

LISTA DE OBSERVACIONES DE TRABAJO DE PARTO Y EXPULSIÓN

No.	PREGUNTA/ ACCIÓN	CÓDIGO DE RESPUESTA	SALTAR A
	**SECCIÓN 6: REVISIÓN DE LA OBSERVACIÓN DE LABOR Y PARTO**		
△	EN CASO NECESARIO, REVISAR EL EXPEDIENTE CLÍNICO/ PREGUNTAR AL PROVEEDOR DESPUÉS DE LA OBSERVACIÓN		
600	¿El proveedor completó un registro de la atención materna / neonatal?	①.SÍ ②.NO ⑧.NO SABE	
601	¿El proveedor utilizó un partograma?	①.SÍ ②.NO ⑧.NO SABE	②→603
602	El partograma no se utilizó debido a que la mujer llegó al hospital en la segunda etapa del parto	①.SÍ ②.NO ⑧.NO SABE	
603	OBSERVADOR: ¿vio si el proveedor llenó el partograma después del parto? (con información que debió completar durante el trabajo de parto)	①.SÍ ②.NO ⑧.NO SABE	
604	Registro del estado/ Resultado de la madre:<br>a. Enviada a sala de recuperación<br>b. Referida a un especialista en el mismo hospital<br>c. Entra a cirugía en el mismo hospital<br>d. Referida a otro hospital<br>e. Muerte materna	①. SALA DE RECUPERACIÓN<br>②. REFERIDA, MISMO HOSP.<br>③. CIRUGÍA<br>④. REFERIDA, OTRO HOSP.<br>⑤. FALLECIMIENTO	
605	Registro del estado/ Resultado del bebé:<br>a. Enviada a cunero normal<br>b. Referido a un especialista en el mismo hospital<br>c. Referido a otro hospital<br>d. Enviado a la habitación con su madre<br>e. Muerte neonatal<br>f. Óbito reciente<br>g. Óbito macerado	①. CUNERO<br>②. REFERIDO, MISMO HOSP.<br>③. REFERIDO, OTRO HOSP.<br>④. ALOJAMIENTO C/MADRE<br>⑤. MUERTE NEONATAL<br>⑥. ÓBITO RECIENTE<br>⑦. ÓBITO MACERADO	
606	Registre el tipo de nacimiento	①. VAGINAL<br>②. ASISTIDO, FORCEPS<br>③. ASISTIDO, VENTOSA<br>④. CESÁREA<br>⑤. NO SABE	①→611<br>②→611<br>③→611<br><br>⑤→611
607	¿La cesárea se realizó antes o después de iniciado el trabajo de parto?	①. ANTES DE INICIADO TdP<br>②. DESPUÉS DE INICADO TdP<br>③. NO SABE	
608	¿Cuál fue la indicación para realizar la cesarea?	①. SANGRADO EXCESIVO<br>②. PARTO OBSTRUIDO<br>③. PARTO PROLONGADO<br>④. RUPTURA UTERINA<br>⑤. MALA PRESENTACIÓN<br>⑥. SUFRIMIENTO FETAL<br>⑦. OTRO<br>ESPECIFICAR: ....<br>⑧. NO HUBO RAZÓN MÉDICA<br>⑨. NO SABE	
609	¿En qué momento se tomó la decisión de realizar una cesárea?	①. ANTES DE INICIADO EL TdP<br>②. DESPUÉS DE INICIADO EL TdP<br>③. NO SABE	

ID PACIENTE [___|___] [___|___] [___|___][___]

ANEXO I **LISTA DE OBSERVACIONES DE TRABAJO DE PARTO Y EXPULSIÓN**

No.	PREGUNTA/ ACCIÓN	CÓDIGO DE RESPUESTA	SALTAR A
△	**SECCIÓN 6: REVISIÓN DE LA OBSERVACIÓN DE LABOR Y PARTO**<br>EN CASO NECESARIO, REVISAR EL EXPEDIENTE CLÍNICO/ PREGUNTAR AL PROVEEDOR DESPUÉS DE LA OBSERVACIÓN		
610	¿Quién decidió que se realizaría una cesárea?	①. DOCTOR/ PROVEEDOR<br>②. MUJER<br>③. NO SABE	
611	Registre si la madre tuvo alguna de la siguientes complicaciones:<br>a. Preeclampsia/eclampsia<br>b. Hemorragia<br>c. Obstrucción del canal de parto<br>d. Parto prolongado<br>e. Ruptura uterina<br>f. Infección puerperal	①. PE/E<br>②. HEMORRAGIA<br>③. OBSTRUCCIÓN<br>④. PARTO PROLONGADO<br>⑤. RUPTURA UTERINA<br>⑥. SEPSIS<br>⑦. OTRA<br>ESPECIFICAR: ....<br>⑧. NINGUNA COMPLICACIÓN<br>⑨. NO SABE	⑨→613
612	¿En qué etapa del trabajo de parto ocurrió la complicación?	①. ANTES DEL PARTO<br>②. DURANTE EL PARTO<br>③. POST PARTO<br>④. NO SABE	
613	¿Se administraron hemoderivados para manejo de hemorragia durante el trabajo de parto, parto o puerperio inmediato?	①. SÍ<br>②. NO<br>③. NO SABE	
614	¿Solicitó la mujer algún medicamento para el manejo del dolor durante el trabajo de parto, parto o puerperio inmediato?	①. SÍ<br>②. NO<br>③. NO SABE	
615	¿Recibió la mujer algún medicamento para el manejo del dolor durante el trabajo de parto, parto o puerperio inmediato?	①. SÍ<br>②. NO<br>③. NO SABE	
616	¿Recibió la mujer algún antibiótico durante el trabajo de parto, parto o puerperio inmediato?	①. SÍ<br>②. NO<br>③. NO SABE	
617	**Hora en que finaliza la observación de labor y parto**<br>✎ Utilice el reloj de 24 horas	HORAS [___|___]<br>MINUTOS [___|___]	
618	NOTAS ESCRITAS DEL (DE LA) OBSERVADOR(A).<br>En particular, comentar sobre lo siguiente:<br>• ¿La mujer fue tratada con respeto? ¿Se le informó de los procedimientos para ella y para su bebé?<br>• ¿Cómo describiría la situación: caótica o calmada y ordenada?<br>• ¿Hubieron demoras para recibir tratamiento/manejo necesario? En caso afirmativo, ¿para qué tipo de procedimientos (madre o bebé) y por qué?¿Estuvieron involucrados múltiples proveedores de salud? ¿Quiénes?<br>• En caso de que el bebé haya muerto, ¿se dio consejería a la madre?<br>• En caso de que no se haya completado la observación, explique por qué.		

....

....

No.	PREGUNTA/ ACCIÓN	CÓDIGO DE RESPUESTA	SALTAR A
△	**SECCION 6: REVISION DE LA OBSERVACION DE LABOR Y PARTO**<br>EN CASO NECESARIO, REVISAR EL EXPEDIENTE CLINICO/ PREGUNTAR AL PROVEEDOR DESPUES DE LA OBSERVACION		
618	NOTAS ESCRITAS DEL OBSERVADOR.<br>En particular, comentar sobre lo siguiente:<br>• ¿La mujer fue tratada con respeto? ¿Se le informó de los procedimientos para ella y para su bebé?<br>• ¿Cómo describiría la situación: caótica o calmada y ordenada?<br>• ¿Hubieron demoras para recibir tratamiento/manejo necesario? En caso afirmativo, ¿para qué tipo de procedimientos (madre o bebé) y por qué?¿Estuvieron involucrados múltiples proveedores de salud? ¿Quiénes?<br>• En caso de que el bebé haya muerto, ¿se dio consejería a la madre?<br>• En caso de que no se haya completado la observación, explique por qué.		

....
....
....
....
....
....
....
....
....
....
....
....
....
....
....
....
....
....
....
....
....
....
....
....
....
....
....
....
....

619	FIN
