# Supplementary material for: Measuring progress in maternal and newborn health care in Mexico: validating indicators of health system contact and quality of care
Source: BMC Pregnancy Childbirth. 2016 Aug 30;16(1):255. doi: 10.1186/s12884-016-1047-0 (PMC5006493; doi:10.1186/s12884-016-1047-0)
Supplement: Additional file 5: Table S1. — List of Maternal and Newborn Care Indicators Assessed and Reported Prevalence. (DOC 173 kb) [file 12884_2016_1047_MOESM5_ESM.doc]

**Additional File 5.**

Table S1**.** List of Maternal and Newborn Care Indicators Assessed and Reported Prevalence^†^.

| **Indicator** | | **Question Item(s) in Client Questionnaire** | **N**  **(Total=**  **597)** | **Women’s Self-Reported Prevalence**  (%) | **Observer Reported (‘True’) Prevalence**  (%) | **Sufficient N for analysis?^#^** |
| --- | --- | --- | --- | --- | --- | --- |
| **Initial Client Assessment** | |  |  |  |  |  |
| Type of facility where gave birth (public hospital) | | Can you tell me the type of facility where you gave birth to your baby (public or private sector)? [*Probe to identify the type of source. If unable to determine whether public or private sector, write the name of the place, according to the woman*] | 464 | 85.2 | 100.0 | N |
| HIV status checked | | During your time at the health facility for the birth of your baby, did someone ask you what your HIV status was? | 569 | 54.1 | 69.8 | Y |
| Offered HIV test | | While you were at the health facility for the birth of your baby, did someone offer you an HIV test? | 559 | 14.3 | 1.8 | N |
| Receives HIV test | | While you were at the health facility for the birth of your baby, did you receive an HIV test? | 429 | 16.3 | 1.2 | N |
| Provider washes hands with soap and water or uses antiseptic before any initial examination | | Did the health provider(s) wash his/her hands with soap and water or use antiseptic before examining you? | 458 | 95.9 | 2.0 | N |
| Takes blood pressure | | Did someone take your blood pressure, at or near your admission to the facility? | 582 | 98.8 | 97.8 | N |
| Takes urine sample | | Did someone ask you to give them a urine sample, at or near your admission to the facility? | 572 | 24.3 | 25.2 | Y |
| Checks fetal heart rate /perform ultrasound | | Did someone listen to the heartbeat of the baby, at or near the admission to the facility? [Or] Did you receive an ultrasound test, at or near your admission to the facility? | 586 | 97.8 | 97.8 | N |
| Wears high-level disinfected or sterile gloves for vaginal examination | | Did the health provider(s) wear rubber gloves during vaginal examinations? | 578 | 100.0 | 99.7 | N |
| **Provider Respectful Care** | |  |  |  |  |  |
| Encourages/assists woman to ambulate during labor | | Were you allowed to get up and walk around while you were in labor? | 580 | 8.3 | 22.2 | Y |
| Woman allowed to drink liquids/eat | | Were you allowed to drink liquids or eat any food while you were in labor? | 590 | 2.9 | 0.5 | N |
| Woman allowed to have a support person present during labor and delivery | | Were you allowed to have a companion with you during your labor or delivery? | 578 | 0.4 | 0.0 | N |
| Encourages woman to assume different positions in labor | | Did a health care provider ask you what position you preferred during labor? | 590 | 3.9 | 67.6 | N |
| A support person is present at some point during labor or delivery | | Were you allowed to have a companion with you during labor or the birth of your baby? | 587 | 0.2 | 0.9 | N |
| A support person is present during labor or delivery | | Did you have a companion with you during labor or delivery? | 589 | 0.2 | 1.0 | N |
| **First Stage of Labor** | |  |  |  |  |  |
| Induces by uterotonic (IV line, IM injection, or tablet) ^a^ | | (1) For the birth of your baby, did your labor start spontaneously or did someone do something to bring on your labor? [AND]  (2) For your birth of your baby, what was done to bring on your labor? [IV line inserted in arm, Rupture membranes, Other] | 559 | 12.9 | 11.6 | Y |
| (Of women whose labor was induced) Uterotonic route for induction of labor by IV line ^a^ | | (1) For the birth of your baby, did your labor start spontaneously or did someone do something to bring on your labor? [Yes]  (2) For your birth of your baby, what was done to bring on your labor? [IV line inserted in arm] | 65 | 27.7 | 100.0 | N |
| Augments labor with uterotonic (by IV line, IM injection, or tablet) ^a^ | | (1) For your delivery, was anything done to speed up or to strengthen your labor? [And]  (2) What was done to speed up or to strengthen your labor? [Injection in thigh/buttocks, IV line inserted in arm, Rupture members, Other] | 472 | 42.0 | 75.2 | Y |
| (Of women whose labor was augmented) Augmentation of labor by IV line ^a^ | | (1) For your delivery, was anything done to speed up or to strengthen your labor? (Yes) [And]  (2) What was done to speed up or to strengthen your labor? [IV line inserted in arm] | 255 | 57.3 | 100.0 | N |
| Receives injection or IV medication during labor (to induce or augment labor) ^a^ | | *‘Yes’ to received injection or IV line for induction [Or] augmentation of labor indicator.* | 476 | 49.8 | 76.1 | Y |
| Injection or IV medication received at some time during labor, before birth of baby (general) ^a^ | | For your delivery, did you receive any injections or IV medication during labor, that is, before the birth of your baby? | 571 | 63.6 | 76.0 | Y |
| Membranes ruptured (to induce or augment labor) | | (1) For your birth of your baby, what was done to bring on your labor? [Membranes ruptured] [Or]  (2) What was done to speed up or to strengthen your labor? [Membranes ruptured] | 356 | 27.0 | 57.0 | Y |
| **Skilled Birth Attendance** | |  |  |  |  |  |
| Skilled main provider labor | | Who was the main provider assisting you during labor? [Doctor (ob-gyn or general), Medical resident, or Nurse] | 580 | 95.7 | 96.7 | N |
| Main provider labor- doctor or medical  resident | | Who was the main provider assisting you during labor? [Doctor (ob-gyn or general) or Medical resident] | 580 | 81.9 | 96.2 | Y |
| Main provider labor- doctor (any) | | Who was the main provider assisting you during labor? [Doctor (ob-gyn or general)] | 580 | 76.7 | 1.7 | N |
| Main provider labor- medical resident | | Who was the main provider assisting you during labor? [Medical resident] | 580 | 5.2 | 94.5 | N |
| Main provider labor- medical intern | | Who was the main provider assisting you during labor? [Medical intern] | 580 | 3.6 | 3.3 | N |
| Main provider labor- nurse | | Who was the main provider assisting you during labor? [Nurse] | 580 | 13.8 | 0.5 | N |
| Main provider labor- nursing student /intern | | Who was the main provider assisting you during labor? [Student nurse or Nurse intern] | 580 | 0.7 | 0.0 | N |
| Skilled main provider delivery | | Who was the main provider assisting you during delivery? [Doctor (ob-gyn or general), Medical resident, or Nurse] | 584 | 97.8 | 92.8 | N |
| Main provider delivery- doctor or medical  resident^†^ | | Who was the main provider assisting you during delivery? [Doctor (ob-gyn or general) or Medical resident] | 584 | 95.7 | 92.5 | N |
| Main provider delivery- doctor (any) | | Who was the main provider assisting you during delivery? [Doctor (ob-gyn or general)] | 584 | 92.8 | 2.4 | N |
| Main provider delivery- medical resident | | Who was the main provider assisting you during delivery? [Medical resident] | 584 | 2.9 | 90.1 | N |
| Main provider delivery- medical student/  intern | | Who was the main provider assisting you during delivery? [Medical intern /student] | 584 | 2.2 | 7.2 | N |
| Main provider delivery- nurse | | Who was the main provider assisting you during delivery? [Nurse] | 584 | 2.1 | 0.3 | N |
| Main provider delivery- nursing student  /intern | | Who was the main provider assisting you during delivery? [Student nurse or Nurse intern] | 584 | 0.0 | 0.0 | N |
| Skilled main provider caught baby | | ho was the main provider who actually delivered the baby (caught the baby)? [Doctor (ob-gyn or general), Medical resident or Nurse] | 563 | 97.0 | 92.2 | N |
| Main provider caught baby- doctor or medical  resident | | Who was the main provider who actually delivered the baby (caught the baby)? [Doctor (ob-gyn or general) or Medical resident] | 563 | 89.7 | 91.1 | Y |
| Main provider caught baby- doctor (any) | | Who was the main provider who actually delivered the baby (caught the baby)? [Doctor (ob-gyn or general)] | 563 | 85.8 | 10.1 | N |
| Main provider caught baby- medical resident | | Who was the main provider who actually delivered the baby (caught the baby)? [Medical resident] | 563 | 3.9 | 81.0 | N |
| Main provider caught baby- medical student/ intern | | Who was the main provider who actually delivered the baby (caught the baby)? [Medical intern/ student] | 563 | 2.3 | 7.8 | N |
| Main provider caught baby- nurse | | Who was the main provider who actually delivered the baby (caught the baby)? [Nurse] | 563 | 7.3 | 1.1 | N |
| Main provider caught baby- nursing student /intern | | Who was the main provider assisting you during delivery? [Student nurse or Nurse intern] | 563 | 0.2 | 0.0 | N |
| **Second & Third Stage of Labor** | |  |  |  |  |  |
| Episiotomy performed | | For your delivery, shortly before you delivered your baby, did anyone cut the opening of your vagina (episiotomy) to make more room for the baby’s head? | 580 | 78.1 | 67.8 | Y |
| Position of mother at birth- on back | | What position were you actually in when you delivered your baby? That is, were you on your back, on your hands and knees, squatting or in another position? [On your back] | 597 | 93.3 | 99.7 | N |
| Did health provider wear gloves during delivery of baby | | Did the health provider(s) wear rubber gloves during delivery of your baby? | 567 | 99.8 | 99.3 | N |
| Uterotonic received 1-3 minutes after delivery | | Immediatel*y* after the birth of your baby, …did anyone give you an injection in your thigh or buttock?  [Or] … did anyone give you medication intravenously (through a tube in your arm)?  [Or] ...did anyone give you tablets to swallow or hold in your mouth?  [Or] ...did anyone place tablets in your rectum? | 580 | 60.7 | 64.0 | Y |
| Uterotonic received after delivery of placenta | | After the birth of your baby, in the first few minutes after the delivery of the placenta, …did anyone give you an injection in your thigh?  [Or] ...did anyone give you tablets to take or hold in your mouth?  [Or] …did anyone give you an injection in your thigh? | 576 | 94.1 | 20.0 | Y |
| Method of uterotonic administration- IV line ^a^ | | Immediately after the delivery of your baby, did anyone give you medication intravenously (through a tube in your arm)? | 498 | 62.2 | 100.0 | N |
| Oxytocin given following delivery | | Immediately after the birth of your baby, did anyone give you a medication or injection called oxytocin to help your uterus contract/become firm? | 358 | 50.0 | 99.2 | N |
| Applies controlled cord traction | | After the delivery of your baby, did the birth attendant help you deliver the placenta, that is, did s/he place his/her hand firmly on your lower abdomen with one hand and hold the umbilical cord in the other hand? | 583 | 93.8 | 96.9 | N |
| Performs uterine massage after birth | | After the delivery of your baby, did the birth attendant firmly massage your lower abdomen to help the womb contract (become firm)? | 573 | 82.4 | 83.1 | Y |
| Palpates uterus after delivery of placenta | | After the delivery of the placenta, did the birth attendant firmly massage your lower abdomen to help the womb contract (become firm)? | 580 | 79.7 | 82.4 | Y |
| 3 AMTSL^b^ elements: prophylactic uterotonic, controlled cord traction, uterine massage post-placenta | | (1) Immediately after the delivery of your baby, …did anyone give you an injection in your thigh or buttock?  [Or] … did anyone give you medication intravenously (through a tube in your arm)?  [Or] ...did anyone give you tablets to swallow or hold in your mouth?  [Or] ...did anyone place tablets in your rectum? [AND]  (2) After the delivery of your baby, did the birth attendant firmly on your lower abdomen with one hand and hold the umbilical cord in the other hand? [AND]  (3) After the delivery of the placenta, did the birth attendant firmly massage your lower abdomen to help the womb contract (become firm)? | 549 | 75.8 | 81.2 | Y |
| **Immediate Postnatal Care for Newborn** ^c^ | |  |  |  |  |  |
| Baby immediately dried with towel or cloth | | Was your baby dried off with a towel immediately after his/her birth, within a few minutes of delivery? | 545 | 99.3 | 93.6 | N |
| Baby given to mother immediately after birth | | Was your baby given to you immediately, within a few minutes, after his/her birth? | 580 | 59.8 | 10.3 | Y |
| Baby placed immediately skin-to-skin on mother's abdomen | | Did someone place the baby on your chest, against your skin, immediately after delivery of the baby? | 581 | 11.4 | 0.2 | N |
| Baby immediately skin-to-skin on mother (2 item)^d^ | | (1) Did someone place the baby on your chest, against your skin, immediately after delivery of the baby? [Yes] [AND]  (2) Was your baby wrapped in a cloth while lying against your chest or was your baby naked against your skin? [Naked against skin] | 574 | 1.1 | 0.2 | N |
| Babies not on skin wrapped with towel | | (1) Did someone place the baby on your chest, against your skin, immediately after delivery of the baby? [No] [AND]  (2) Was your baby wrapped in a cloth while lying against your chest or was your baby naked against your skin? [Wrapped in cloth] | 567 | 99.1 | 98.8 | N |
| Breastfeeding initiated within first hour of birth | | Did you breastfeed your baby within the first hour after delivery? | 449 | 64.8 | 34.7 | Y |
| Something other than breastmilk given to baby within first hour of delivery | | Did you or anyone else give anything to the baby to eat or drink within the first hour after delivery? | 438 | 57.8 | 21.7 | Y |
| Baby bathed within the first hour after birth | | About how long after birth was your baby bathed for the first time? [Within1 hour] | 441 | 0.7 | 2.7 | N |
| 3 elements of newborn care (newborn immediately dried + placed naked on skin + breastfed within first hour) | | (1) Was your baby dried off with a towel or cloth immediately after his/her birth, within a few minutes of delivery? [AND]  (2) Did someone place the baby on your chest, against your skin, immediately after delivery of the baby? [AND]  (3) Did you breastfeed your baby within the first hour after delivery? | 414 | 9.7 | 0.0 | N |
| 3 elements of newborn care (newborn immediately dried + placed naked on skin (2 item)^d^+ breastfed within first hour) | | (1) Was your baby dried off with a towel or cloth immediately after his/her birth, within a few minutes of delivery? [AND] [AND] Was your baby wrapped in a cloth while lying against your chest or was your baby naked against your skin? [Naked against skin]  (2) Did someone place the baby on your chest, against your skin, immediately after delivery of the baby? [AND]  (3) Did you breastfeed your baby within the first hour after delivery? | 407 | 0.7 | 0.0 | N |
| **Immediate Postnatal Care for Mother** | |  |  |  |  |  |
| Provider did at least one post-delivery health check | | In your first physical examination/check after delivery, did a health provider… [Yes to any of below health checks] | 593 | 100.0 | 99.3 | N |
| In first post-delivery exam, checks for bleeding | | In your first physical examination/check after delivery, did a health provider look for or ask you about bleeding? | 590 | 87.5 | 80.5 | Y |
| In first post-delivery exam, examines perineum | | In your first physical examination/check after delivery, did a health provider do a perineal exam? | 586 | 90.1 | 75.1 | Y |
| In first post-delivery exam, takes temperature | | In your first physical examination/check after delivery, did someone take your temperature? | 586 | 93.3 | 66.9 | Y |
| In first post-delivery exam, takes blood pressure | | In your first physical examination/check after delivery, did someone take your blood pressure? | 589 | 93.4 | 95.6 | Y |
| In first post-delivery exam, checks for involution | | In your first physical examination/check after delivery, did a health provider check your belly to see if your womb was becoming firm after the birth of your baby? | 556 | 83.5 | 81.7 | Y |
| Woman asked for pain relief medication while at facility | | Did you ask for pain relief at any point during your time at the health facility? | 575 | 26.3 | 44.7 | Y |
| Woman received pain relief medication | | Did anyone give you any pain relief at any point during your time at the health facility? | 581 | 87.4 | 79.4 | Y |
| **Maternal and Newborn Outcomes** | |  |  |  |  |  |
| Complications (any) | | Did you experience any of the following complications during or within the first few hours after your delivery? [Yes to any of below symptoms] | 595 | 38.2 | 13.6 | Y |
| High blood pressure/ convulsions (eclampsia) | | Did you experience any of the following complications during or after your delivery?  [(1) High blood pressure, seizures, blurred vision, severe headaches  [Or] (2) Swelling in hands or feet] | 594 | 21.4 | 2.4 | Y |
| Severe bleeding (hemorrhage) | | Did you experience any of the following complications during or within the first few hours after your delivery? [Excessive bleeding] | 593 | 8.4 | 8.1 | Y |
| Prolonged labor (>12 hours) | | Did you experience any of the following complications during or within the first few hours after your delivery? [Long labor, more than 12 hours] | 595 | 7.4 | 2.2 | N |
| Prolonged/obstructed labor | | Did you experience any of the following complications during or within the first few hours after your delivery? [(1) Long labor, more than 12 hours [Or] (2) Baby in distress/ too large] | 595 | 10.8 | 3.0 | N |
| None | | Did you experience any of the following complications during or within the first few hours after your delivery? [None] | 595 | 61.9 | 86.4 | Y |
| Blood products given | | Did you receive a blood transfusion at any point during your delivery or stay at the facility? | 582 | 2.4 | 2.4 | Y |
| Low birth-weight baby (<2,500 g) | | What was your baby’s weight? [weight given in grams <2,500 g)] | 589 | 12.1 | 11.2 | N |
| High birth-weight baby (>=4,500 g) | | What was your baby’s weight? [weight given in grams >=4,500 g] | 589 | 0.0 | 0.0 | N |
| Stillborn delivery | | What was the outcome for your delivery? Was your baby born alive or stillborn? | 591 | 0.3 | 0.3 | N |
|  | ^†^ Table presents descriptive results. Validation analysis presented in text was restricted to matched data and excluded ‘Don’t Know’ and missing responses; ^#^At least 5 counts per cell of 2 by 2 table.  ^a^ IV refers to intravenous medication; IM refers to intramuscular injection.  ^b^ AMSTL refers to active management of the third stage of labor.  ^b^ Questions asked for mothers who delivered baby that was breathing at time of birth.  ^c^ Indicator constructed from two skin-to-skin items: (1) baby placed against mother’s chest after delivery and (2) baby was naked against the mother’s chest. | | | | | |
